# Supplementary figures and images for: Carbohydrate sulfotransferase 14 gene deletion induces dermatan sulfate deficiency and affects collagen structure and bowel contraction
Source: PLoS One. 2025 May 6;20(5):e0320943. doi: 10.1371/journal.pone.0320943 (PMC12054877; doi:10.1371/journal.pone.0320943)

**A**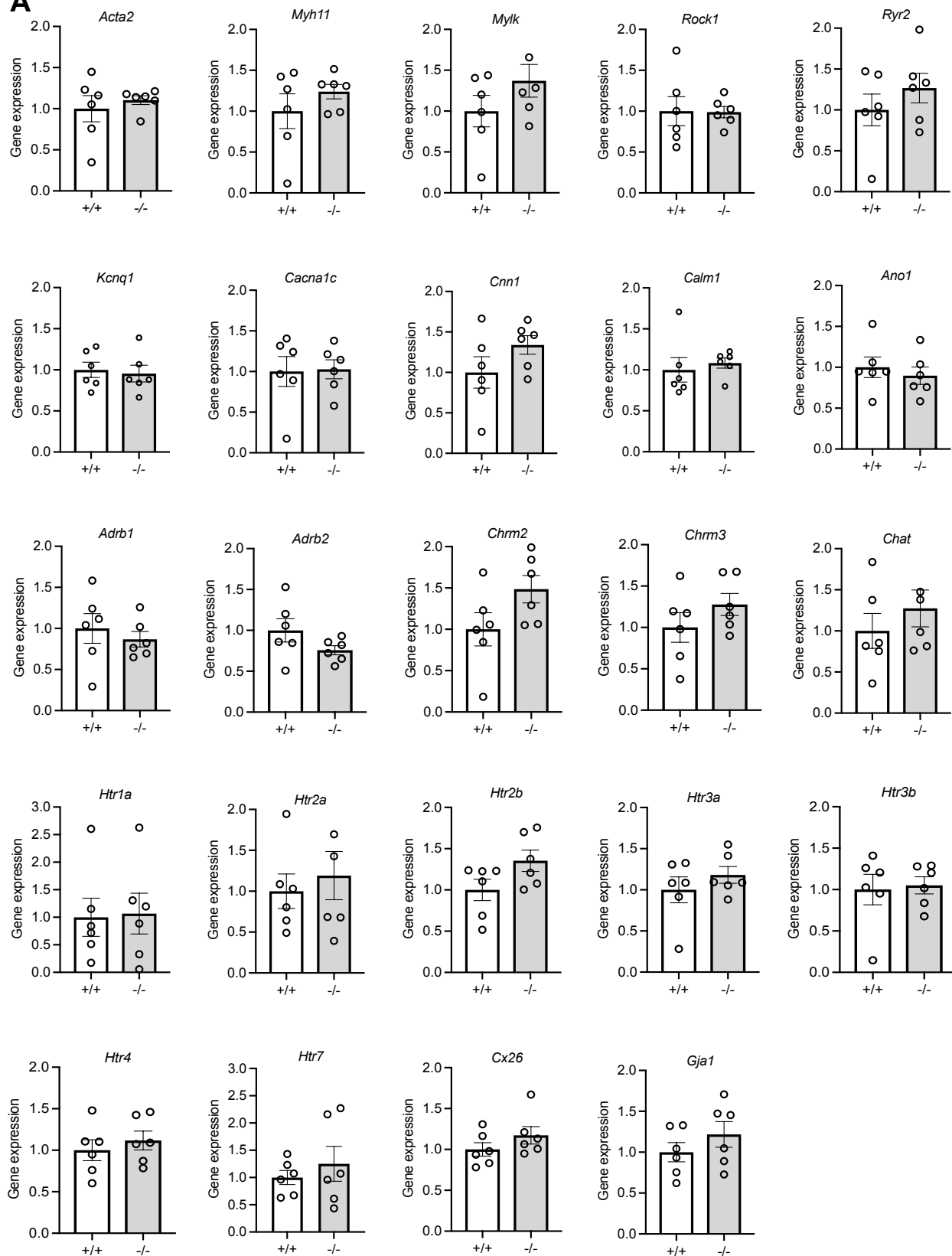

Supplement: S1 Fig — (A) Relative mRNA levels (mean ± SEM) of Acta2, Myh11, Mylk, Rock1, Ryr2, Kcnq1, Cacna1c, Cnn1, Calm1, Ano1, Adrb1, Adrb2, Chrm2, Chrm3, Chat, Htr1a, Htr2a, Htr2b, Htr3a, Htr3b, Htr4, Htr7, Cx26, and Gja1 in colon samples from 10-week-old Chst14+/+ and Chst14-/- mice (n = 6 per group). Gene abbreviations: Acta2, actin alpha 2; Myh11, myosin heavy chain 11; Mylk, myosin light chain kinase; Rock1, Rho-associated cooled containing protein kinase 1; Ryr2, ryanodine receptor 2; Kcnq1, potassium voltage-gated channel subfamily Q member 1; Cacna1c, calcium channel voltage-dependent L type alpha 1C subunit; Cnn1, calponin 1; Calm1, calmodulin 1; Ano1, calcium activated chloride channel; Adrb1, adrenergic receptor, beta 1; Adrb2, adrenergic receptor, beta 2; Chrm2, cholinergic receptor, muscarinic 2; Chrm3, cholinergic receptor, muscarinic 3; Chat, choline acetyltransferase; Htr1a, 5-hydroxytryptamine receptor 1A; Htr2a, 5-hydroxytryptamine receptor 2A; Htr2b, 5-hydroxytryptamine receptor 2B; Htr3a, 5-hydroxytryptamine receptor 3A; Htr3b, 5-hydroxytryptamine receptor 3B; Htr4, 5-hydroxytryptamine receptor 4; Htr7, 5-hydroxytryptamine receptor 7; Cx26, connexin-26; Gja1, gap junction protein alpha 1. (PDF) [file pone.0320943.s001.pdf]

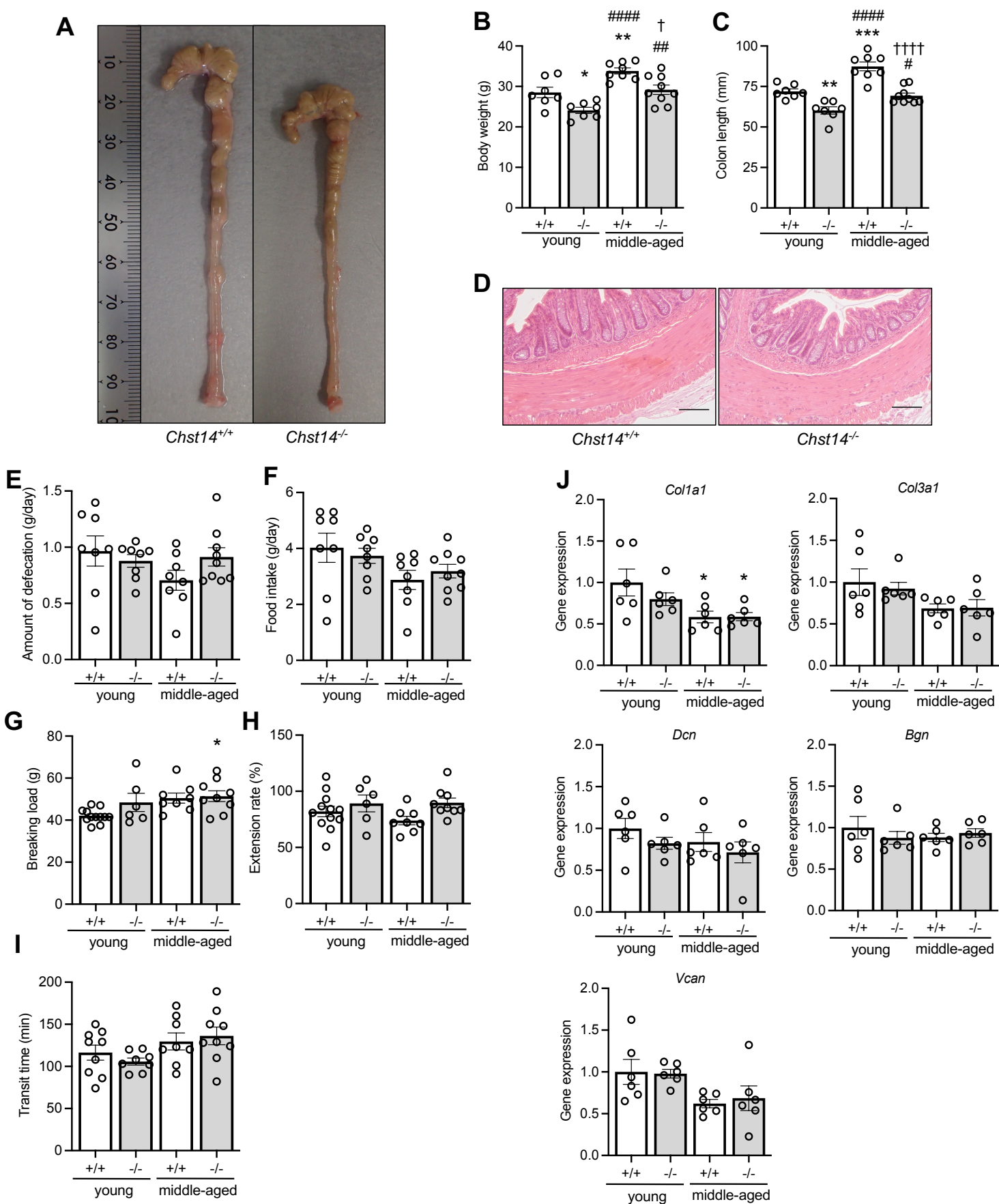

Supplement: S2 Fig — (A) Typical appearance of the colon in middle-aged mice. (B) Body weight (mean ± SEM). (C) Comparison of colon length between young and middle-aged groups (mean ± SEM). (B), (C) Young Chst14+/+ mice, n = 7; and Chst14-/- mice, n = 7; and middle-aged Chst14+/+ mice, n = 8; and Chst14-/- mice, n = 9. Data were analyzed using one-way ANOVA followed by Tukey–Kramer post hoc test. *P < 0.05, **P < 0.01, ***P < 0.001 compared with young Chst14+/+ group. ##P < 0.01, ####P < 0.0001 compared with young the Chst14-/- group. †P < 0.05, ††††P < 0.0001 compared with middle-aged Chst14+/+ group. (D) H&E-stained colon sections from middle-aged Chst14+/+ and Chst14-/- mice. Scale bar: 200 µm. (E) Amount of defecation per day. (F) Daily food intake. (E, F) Young Chst14+/+ mice, n = 8; and young Chst14-/- mice, n = 8; and middle-aged Chst14+/+ mice, n = 8; and middle-aged Chst14-/- mice, n = 9. (G) Load required for colon rupture. (H) Rate of colon extension when loaded with a 10 g weight. (G, H) Young Chst14 +/+ mice, n = 12; and young Chst14-/- mice, n = 6; and middle-aged Chst14+/+ mice, n = 8; and middle-aged Chst14-/- mice, n = 9. (I) Gastrointestinal transit time. Young Chst14+/+ mice, n = 9; and young Chst14-/- mice, n = 8; and middle-aged Chst14+/+ mice, n = 8; and middle-aged Chst14-/- mice, n = 9. (J) Relative mRNA levels of Col1a1, Col3a1, Dcn, Bgn, and Vcan in the colon (mean ± SEM) analyzed by one-way ANOVA followed by Tukey–Kramer post hoc test. Young and middle-aged Chst14+/+ and Chst14-/- mice (n = 6 per group). Young mice, 10-week-old male mice; middle-aged mice, 36- to 40-week-old male mice. Chst14+/+ mice, + / + ; Chst14-/- mice, -/-. (PDF) [file pone.0320943.s002.pdf]

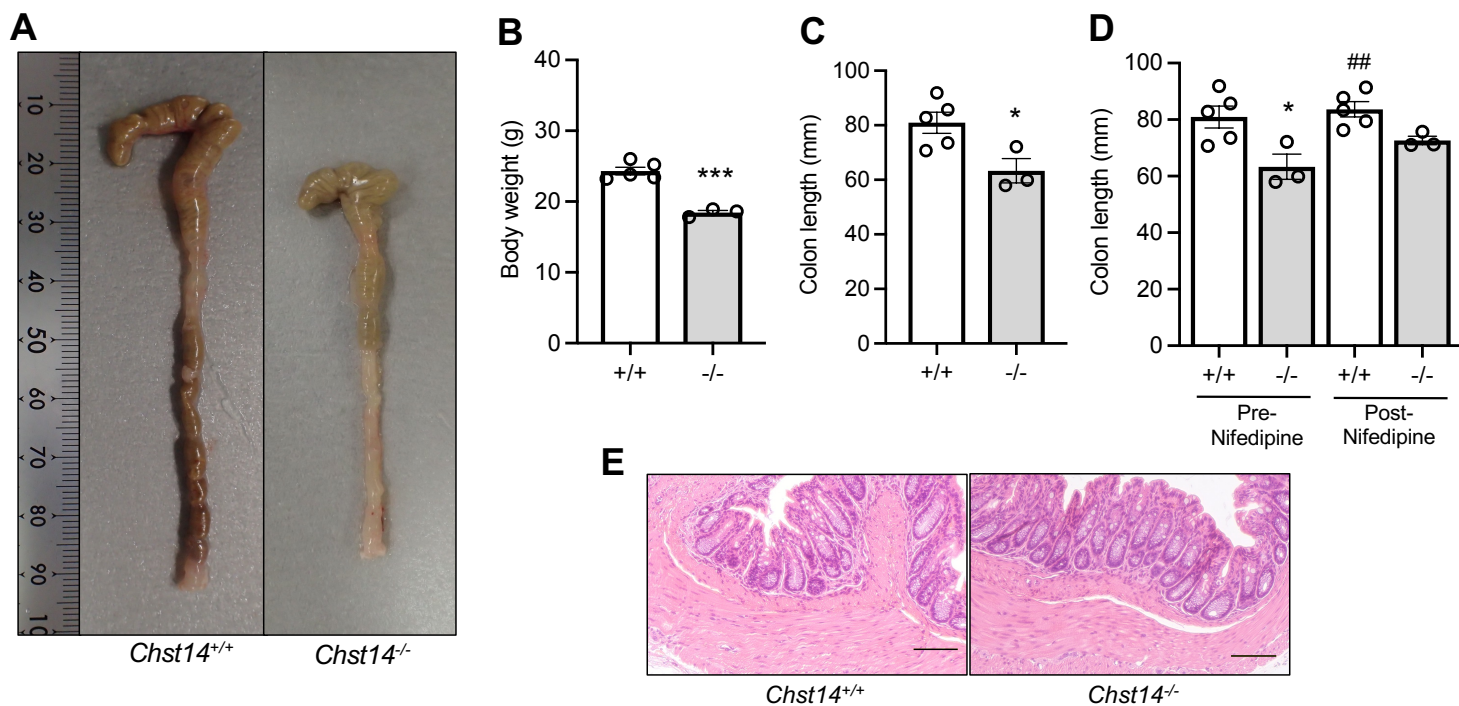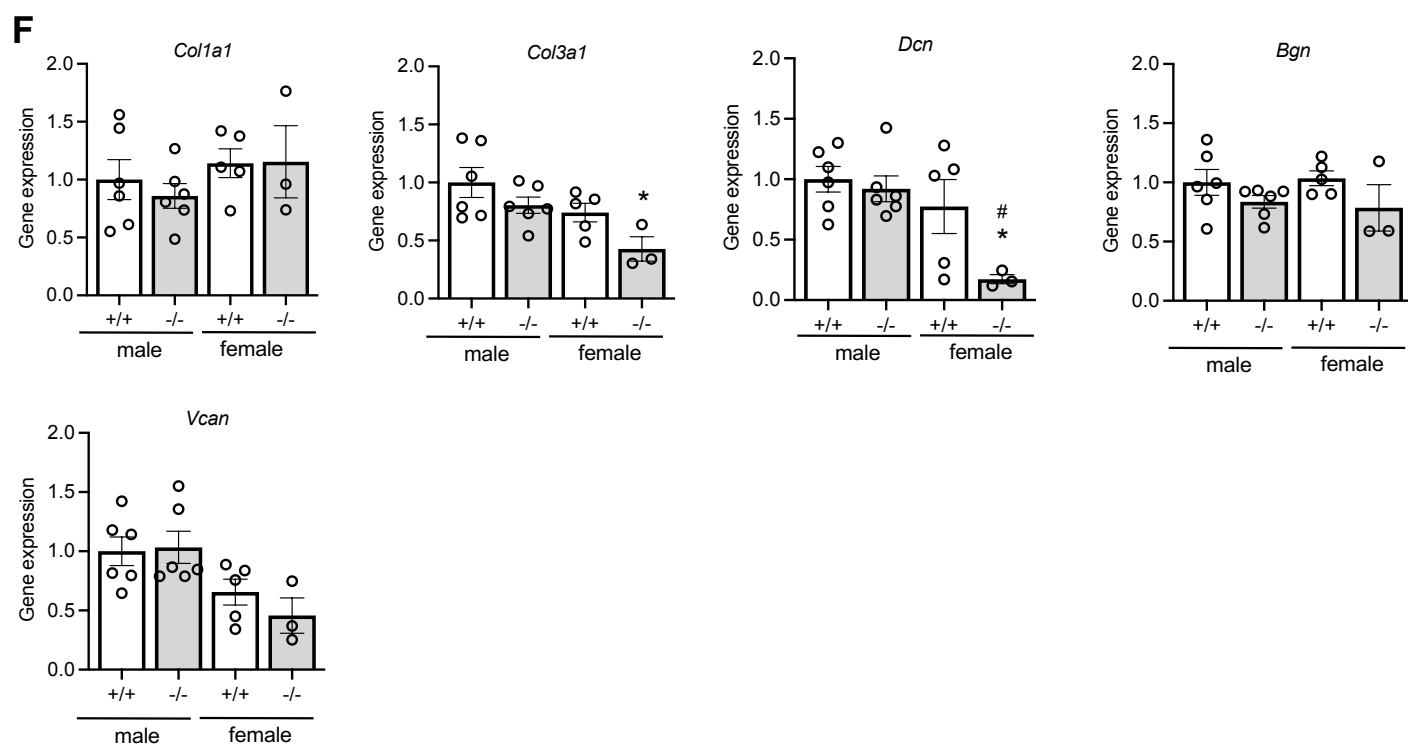

Supplement: S3 Fig — (A) Typical appearance of the colon in female mice. (B) Comparison of body weight between female Chst14+/+ and Chst14-/- mice. (mean ± SEM). ***P < 0.001 compared with Chst14+/+. (C) Comparison of colon length between female Chst14+/+ and Chst14-/- mice. (mean ± SEM). *P < 0.05 compared with Chst14+/+. (B, C) Chst14+/+ mice, n = 5 and Chst14-/- mice, n = 3. Data were analyzed using t-test. (D) Comparison of colon length between pre- and post-nifedipine groups (mean ± SEM). Pre- and post-nifedipine Chst14+/+ mice (n = 5) and Chst14-/- mice (n = 3). Data were analyzed using one-way ANOVA followed by Tukey–Kramer post hoc test. *P < 0.05 compared with pre-nifedipine Chst14+/+ group. ##P < 0.001 compared with pre-nifedipine Chst14-/- group. (E) H&E-stained colon sections from Chst14+/+ and Chst14-/- mice. Scale bar: 200 µm. (F) Relative mRNA levels of Col1a1, Col3a1, Dcn, Bgn, and Vcan in the colon (mean ± SEM) analyzed by one-way ANOVA followed by Tukey–Kramer post hoc test. Male and female Chst14+/+ and Chst14-/- mice (n = 6 per group). *P < 0.05 compared with male Chst14+/+ group. #P < 0.05 compared with male Chst14-/- group. Chst14+/+ mice, + / + ; Chst14-/- mice, -/-; Male, 10-week-old male mice; female, 12-week-old female mice. (PDF) [file pone.0320943.s003.pdf]

**A**

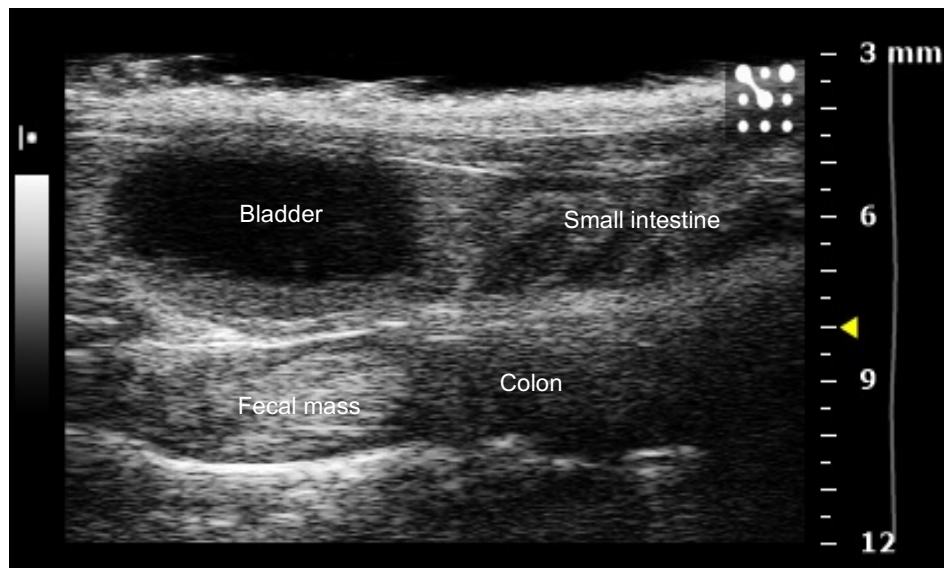

*Chst14*<sup>+/+</sup>

**B**

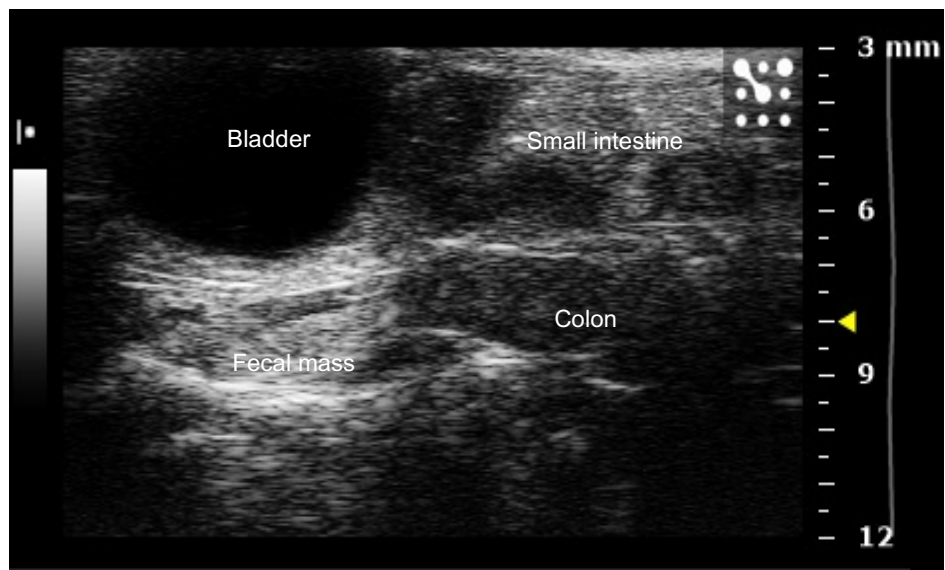

*Chst14*<sup>-/-</sup>

Supplement: S4 Fig — (PDF) [file pone.0320943.s004.pdf]

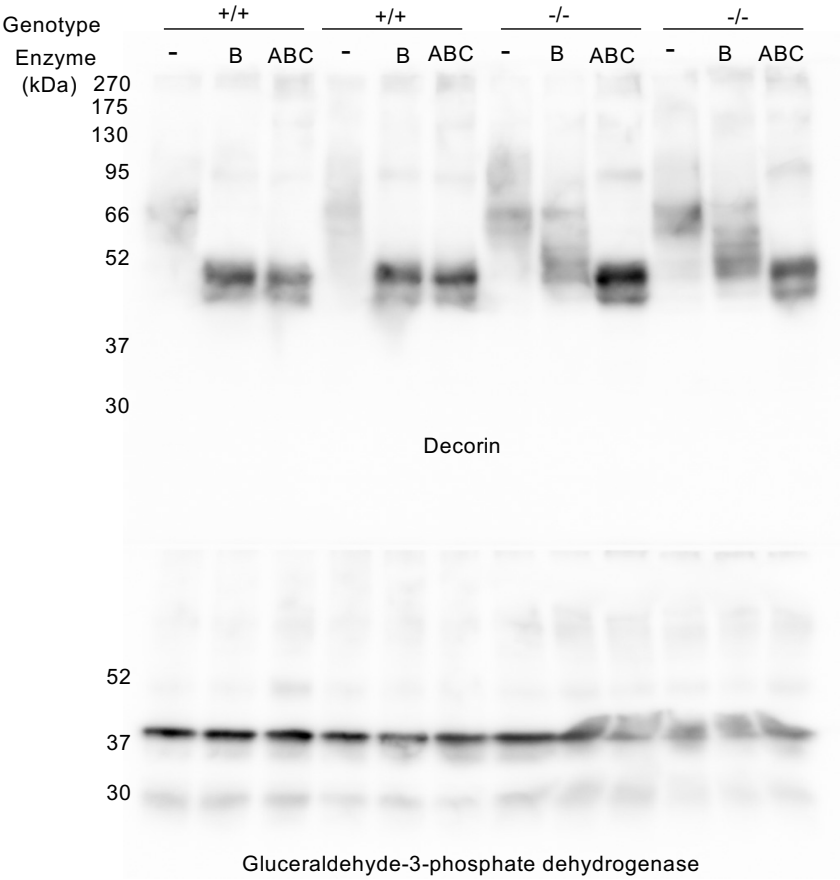

Supplement: S1 File — Uncropped Western blot images for DCN and GAPDH. (PDF) [file pone.0320943.s015.pdf]
