# Supplementary material for: Carbohydrate sulfotransferase 14 gene deletion induces dermatan sulfate deficiency and affects collagen structure and bowel contraction
Source: PLoS One. 2025 May 6;20(5):e0320943. doi: 10.1371/journal.pone.0320943 (PMC12054877; doi:10.1371/journal.pone.0320943)
Supplement: S1 Table — (PDF) [file pone.0320943.s007.pdf]

RT-qPCR primer sequences

Gene	F/R	Sequence (5'–3')
18s Ribosomal RNA	F	ATCATGCAGAACCCACGACA
	R	ACACGAAGGCCCCAAAAGT
Chst3	F	TGACTCAGTTCCTGTTCCGC
	R	CGGTTCCTGCAGTGGTACTT
Chst11	F	CCCTTCGGTGTGGACATCTG
	R	GCAGGATGGCAGTGTTGGAT
Chst12	F	TTCTGACGCAAAGCACAACG
	R	AGGGTTCTGATGGGCATCATG
Chst14	F	CCAAAGTGGCCTGCTCTAACTG
	R	AAGTCACTGCGGTGGTCCAT
Chst15	F	CCAGCAAATTCCTCCCGACT
	R	AACCGCTTGGAGTAGAGCAC
Dse	F	AAGATGCGCCTTGGGATGAA
	R	TCCTGCTGTGTCTTGCTCAG
Ust	F	TTCACAACAAGACCCGGCTT
	R	TAGACAGGCTGGTCTCCTCC
Col1a1	F	ATGGATTCCCGTTCGAGTACG
	R	TCAGCTGGATAGCGACATCG
Col3a1	F	TGACTGTCCCACGTAAGCAC
	R	AGGAAGATCAGGAGGGCCAT
Bgn	F	GAGGGAACTTCACTTGGACAAC
	R	CACCTTGGTGATGTTGTTGG
Dcn	F	ATCACCAAGCTGCGGAAATC
	R	AAGGCTCCGTTTTCAATCCC
Vcan	F	GTTCCACATCACTGCTCCCA
	R	AGTTCTCCAACAGTCGCCAG
Tgfb	F	CCCGAAGCGGACTACTATGC
	R	TAGATGGCGTTGTTGCGGT
Tnfa	F	CCAAATGGCCTCCCTCTCAT
	R	TTGCTACGACGTGGGCTACA
Il1b	F	TCGTGCTGTCGGACCCATAT
	R	GGTTCTCCTTGTACAAAGCTCATG

Gene	F/R	Sequence (5'–3')
Acta2	F	GGCATCCACGAAACCACCTA
	R	AATGCCTGGGTACATGGTGG
Mylk	F	AGCCCAATCAATGCGGAGAA
	R	TCCCTCCACGACTTCCAGAT
Myh11	F	GGGAACAGGAGGTGACAGTG
	R	CCTTGGCCCTTTTGAACTGC
Ryr2	F	GGTCAGCGTGTCCTCAGAAA
	R	TCACCGCCAATGAGATAGCC
Rock1	F	AGTCCTTGGGTTGTTCAGCT
	R	AGGCACGTCATAGTTGCTCA
Ano1	F	ACATCAGCCAGCAGATCCAC
	R	TCCATGTGTCCAGTAGCTGC
Kcnq1	F	GCTGTCCATGCAACAAGGTG
	R	TCAGTTGTTCGTAGGTGGGC
Cacna1c	F	GAGGAGGAGTTGGACAAGGC
	R	AGTAGGTGACATGGTTGCCG
Cnn1	F	CACACTCAACGTCAGCTTGC
	R	TACTCCGGGTTCAGGCAGTA
Calm1	F	GGCACCATTGACTTCCCAGA
	R	TGCCGCACTGATGTAACCAT
Chat	F	CTGGCCACCTACCTTCAGTG
	R	CCCAAACCGCTTCACAATGG
Adrb1	F	TGCTCATCGTGGTGGGTAAC
	R	TGAAGAGGTTGGTGAGCGTC
Adrb2	F	GCCAAACACTTGTCAGCTGG
	R	CTTCCTTGGGAGTCAACGCT
Chrm2	F	AGTGGGATCGTCAGGTCAGA
	R	GAGGCTTCTTTTTGGCAGGC
Chrm3	F	AAGGCACGAAACGGTCATCT
	R	CTTGGTCCATCTGCTCAGCA
Htr1a	F	TACTCCACTTTCGGCGCTTT
	R	CGCTCCCTTCTTTTCCACCT

Gene	F/R	Sequence (5'–3')
Htr2a	F	GAACCAACCTCTCCTGCGAA
	R	GGACACTGCCATGATGACCA
Htr2b	F	TGCTTATGTGGTGCCCCTTT
	R	ATCAGAGGATTCACCCCCGA
Htr3a	F	TTCTACGTGATCATCCGCCG
	R	AGAGACTCTCTCACCGCTGT
Htr3b	F	ACTCTGATGCCGAGGAGTCT
	R	TGTTGATGGACCGGAACTGG
Htr4	F	TGTGTCATCATGGGCTGCTT
	R	TAGCCAAGCCAGAGGAAAGC
Htr7	F	AAGTTCTCAGGCTTCCCACG
	R	TTCGCACACTCTTCCACCTC
Cx26	F	TCCGGGTCATCTTTGAAGCC
	R	CCAAGCGTTGCATTTCACCA
Gja1	F	TGGGGTGATGAACAGTCTGC
	R	ACGTGAGAGATGGGGAAGGA
