# Supplementary material for: Carbohydrate sulfotransferase 14 gene deletion induces dermatan sulfate deficiency and affects collagen structure and bowel contraction
Source: PLoS One. 2025 May 6;20(5):e0320943. doi: 10.1371/journal.pone.0320943 (PMC12054877; doi:10.1371/journal.pone.0320943)
Supplement: S3 Table — (PDF) [file pone.0320943.s009.pdf]

		Chst14$^{+/+}$			Chst14$^{-/-}$		
		1	2	3	1	2	3
		pmol disaccharide/mg protein					
CS/DS	ΔHexUA-GalNAc	N.D.	N.D.	N.D.	N.D.	N.D.	N.D.
	ΔHexUA-GalNAc(6S)	32.7	32.8	39.9	95.3	92.6	73.2
	ΔHexUA-GalNAc(4S)	824.5	866.1	929.8	699.4	685.8	613.6
	ΔHexUA(2S)-GalNAc(6S)	N.D.	N.D.	N.D.	N.D.	N.D.	N.D.
	ΔHexUA(2S)-GalNAc(4S)	44.3	33.2	39.7	N.D.	N.D.	N.D.
	ΔHexUA-GalNAc(4S,6S)	30.1	51.4	53.3	41.3	45.3	39.8
	ΔHexUA(2S)-GalNAc(4S,6S)	N.D.	N.D.	N.D.	N.D.	N.D.	N.D.
	Total CS/DS disaccharides	931.7	988.4	1062.6	835.9	823.7	726.6
CS	ΔHexUA-GalNAc	N.D.	N.D.	N.D.	N.D.	N.D.	N.D.
	ΔHexUA-GalNAc(6S)	32.7	32.8	39.9	95.3	92.6	73.2
	ΔHexUA-GalNAc(4S)	410.7	397.7	435.1	699.4	685.8	613.6
	ΔHexUA(2S)-GalNAc(6S)	N.D.	N.D.	N.D.	N.D.	N.D.	N.D.
	ΔHexUA(2S)-GalNAc(4S)	7	0.1	-6.1	0	0	0
	ΔHexUA-GalNAc(4S,6S)	30.1	51.4	53.3	41.3	45.3	39.8
	ΔHexUA(2S)-GalNAc(4S,6S)	N.D.	N.D.	N.D.	N.D.	N.D.	N.D.
	Total CS/DS disaccharides	480.5	482	522.2	835.9	823.7	726.6
DS	ΔHexUA-GalNAc	N.D.	N.D.	N.D.	N.D.	N.D.	N.D.
	ΔHexUA-GalNAc(6S)	N.D.	N.D.	N.D.	N.D	N.D	N.D
	ΔHexUA-GalNAc(4S)	413.8	468.4	494.7	N.D	N.D	N.D
	ΔHexUA(2S)-GalNAc(6S)	N.D.	N.D.	N.D.	N.D.	N.D.	N.D.
	ΔHexUA(2S)-GalNAc(4S)	37.3	33.1	45.8	N.D.	N.D.	N.D.
	ΔHexUA-GalNAc(4S,6S)	N.D.	N.D.	N.D.	N.D	N.D	N.D
	ΔHexUA(2S)-GalNAc(4S,6S)	N.D.	N.D.	N.D.	N.D.	N.D.	N.D.
	Total CS/DS disaccharides	451.1	501.5	540.5	N.D	N.D	N.D
