# Supplementary material for: Carbohydrate sulfotransferase 14 gene deletion induces dermatan sulfate deficiency and affects collagen structure and bowel contraction
Source: PLoS One. 2025 May 6;20(5):e0320943. doi: 10.1371/journal.pone.0320943 (PMC12054877; doi:10.1371/journal.pone.0320943)
Supplement: S4 Table — (PDF) [file pone.0320943.s010.pdf]

Body weight and colon length

Group	Baseline						Nifedipine								Baseline				Nifedipine		Baseline			
Genotype	*Chst14*$^{+/+}$		*Chst14*$^{-/-}$		*Chst14*$^{+/+}$		*Chst14*$^{+/+}$				*Chst14*$^{-/-}$				*Chst14*$^{+/+}$		*Chst14*$^{-/-}$		*Chst14*$^{+/+}$	*Chst14*$^{-/-}$	*Chst14*$^{+/+}$		*Chst14*$^{-/-}$	
Age	10w (male)				6w (male)		10w (male)								12w (female)						middle aged (male)			
	BW (g)	colon (mm)	BW (g)	colon (mm)	BW (g)	colon (mm)	BW (g)	pre_colon (mm)	post_colon (mm)	rate(%)	BW (g)	pre_colon (mm)	post_colon (mm)	rate(%)	BW (g)	colon (mm)	BW (g)	colon (mm)	colon (mm)	colon (mm)	BW (g)	colon (mm)	BW (g)	colon (mm)
1	33.2	76.38	22.7	65.882	23.9	66.586	29	84.901	85.204	0.35688626	25.1	60.946	76.031	24.75141929	23.4	82.799	18.6	59.867	83.364	71.191	36.1	82.345	32.4	65.725
2	27.2	72.461	26.7	61.501	25.1	75.092	30.8	67.51	72.95	8.05806547	28.7	72.447	78.08	7.775339213	23.2	73.548	17.8	57.932	79.476	71.042	34.5	98.345	34.6	65.434
3	28.9	78	25.8	57.819	29.4	83.386	31.4	80.333	87.145	8.47970324	29.8	75.431	78.801	4.467659185	23.8	70.68	18.9	72.133	76.3	75.675	32.7	92.673	32.6	67.259
4	27.7	69.15	25	59.954	26.6	74.257	31.6	74.281	77.305	4.07102758	21.5	67.245	74.283	10.46620567	25.2	85.676			87.704		30.6	91.43	30.1	77.231
5	32.7	70.556	21.1	48.591	24.7	82.272	30.2	74.918	80.568	7.54157879	25.9	74.292	84.279	13.44290099	26.0	91.882			91.377		31.8	74.17	28.3	77.78
6	26.5	70.932	24.8	61.348	24.9	79.747	30.2	80.652	88.762	10.0555473	27.4	72.544	88.261	21.66547199							36.4	92.373	24.5	65.122
7	23.4	66.248	22.5	66.183	25.1	80.096	33.3	74.95	87.581	16.8525684	25.8	67.133	83.753	24.756826							35.6	83.676	24.8	70.844
8					25.8	70.259	33.9	83.907	90.716	8.11493678											32.9	84.131	27.1	69.032
9																							28.2	65.47

Amount of defecation / food intake

Genotype	*Chst14*$^{+/+}$			*Chst14*$^{-/-}$			*Chst14*$^{+/+}$			*Chst14*$^{-/-}$		
Age	7w (male)						middle aged (male)					
	BW (g)	food (g/day)	stool (g/day)	BW (g)	food (g/day)	stool (g/day)	BW (g)	food (g/day)	stool (g/day)	BW (g)	food (g/day)	stool (g/day)
1	27.2	4	0.9543	24.1	4.7	0.9839	36.1	3.5	0.6913	32.6	3.8	1.1209
2	31.5	5.3	1.2916	24.3	4.4	0.9652	33.3	3	0.7427	29.9	3.1	0.8608
3	24.7	4	1.2593	22	4	0.9713	30.7	2.5	0.5571	33	3	0.7426
4	28.2	5.3	1.3969	22.2	3.7	0.9425	29.2	3.4	0.8259	26.1	2.6	0.7024
5	26.7	5	0.9884	24.2	2.5	0.5908	29.3	1	0.2285	29.5	4.4	1.444
6	25.1	2.2	0.5927	22	4.3	1.053	35.2	2.1	0.6152	23.5	2.5	0.701
7	23.8	1.4	0.2603	22.8	3.4	0.7255	33.6	3.7	1.0342	21.6	2.1	0.7257
8	27.7	5	0.9849	17.7	2.9	0.7895	30.7	3.8	0.9475	24.8	3.6	0.998
9										27.2	3.6	0.9276

Expulsion time

Genotype	*Chst14*$^{+/+}$		*Chst14*$^{-/-}$	
Age	10–13w (male)			
	BW (g)	time (sec)	BW (g)	time (sec)
1	27.9	90	28.4	67
2	25.8	82	26.5	88
3	29	58	24.9	42
4	31.1	42	26.9	32
5	27.5	28	27.4	27
6	31.2	48	25.8	45
7	30.8	48		
8	33.3	25		
9	33.9	23		

Transit time

Genotype	*Chst14*$^{+/+}$		*Chst14*$^{-/-}$		*Chst14*$^{+/+}$		*Chst14*$^{-/-}$	
Age	10–11w (male)				middle aged (male)			
	BW (g)	time (min)	BW (g)	time (min)	BW (g)	time (min)	BW (g)	time (min)
1	29.3	129	25.6	108	34.7	123	33.5	124
2	30.7	86	26.3	102	35.8	120	34.7	166
3	30.3	150	28.1	111	33.5	160	37.8	148
4	24.8	93	29.9	103	31.4	91	27.3	129
5	28.6	129	27	91	33.9	172	28.8	128
6	31.1	108	27.7	121	38.7	101	24.5	82
7	23.4	137	25.1	120	35.7	120	24.8	150
8	31	141	22.5	90	33.3	150	27.2	110
9	28.8	74					29.4	189

Breaking test

Genotype	*Chst14*$^{+/+}$		*Chst14*$^{-/-}$		*Chst14*$^{+/+}$		*Chst14*$^{-/-}$	
Age	8–10w (male)				middle aged (male)			
	load (g)	ave (g)	load (g)	ave (g)	load (g)	ave (g)	load (g)	ave (g)
1	41	41.5	41	41	51	52	63	63.5
	42				53		64	
2	44	41.5	43	42.5	51	50.5	59	60
	39		42		50		61	
3	44	41.5	43	46.5	65	64	47	47.5
	39		50		63		48	
4	46	47	53	54.5	55	54.5	51	49.5
	48		56		54		48	
5	42	44.5	36	39	43	45	57	56
	47		42		47		55	
6	38	37.5	66	67	49	48	42	42
	37		68		47		42	
7	41	41.5			42	42	52	53
	42				42		54	
8	45	44			46	48	51	50.5
	43				50		50	
9	43	40.5					42	40.5
	38						39	
10	47	41.5						
	36							
11	40	36.5						
	33							
12	50	47.5						
	45							

Extension test

Genotype	*Chst14*$^{+/+}$				*Chst14*$^{-/-}$				*Chst14*$^{+/+}$				*Chst14*$^{-/-}$			
Age	8–10w (male)								middle aged (male)							
	pre (mm)	post (mm)	extension rate (%)	rate ave (%)	pre (mm)	post (mm)	extension rate (%)	rate ave (%)	pre (mm)	post (mm)	extension rate (%)	rate ave (%)	pre (mm)	post (mm)	xtension rate (%	rate ave (%)
1	5.929	3.255	82.15053763	84.1322287	5.16	3.289	56.88659167	60.37537628	4.382	7.694	75.58192606	79.90982608	3.348	6.795	102.9569892	117.027487
	5.522	2.967	86.11391978		4.97	3.033	63.8641609		3.87	7.13	84.2377261		2.878	6.651	131.0979847	
2	5.279	2.793	89.00823487	102.616633	5.475	3.242	68.87723627	78.9338632	3.533	7.082	100.4528729	92.97883352	3.235	5.526	70.81916538	83.16831703
	5.304	2.453	116.2250306		5.167	2.734	88.99049012		3.546	6.578	85.50479413		3.034	5.932	95.51746869	
3	6.008	3.126	92.19449776	86.7137902	5.583	2.835	96.93121693	110.2962199	4.625	7.244	56.62702703	59.01017534	3.511	6.447	83.62289946	82.54476397
	6.026	3.325	81.23308271		5.889	2.633	123.6612229		4.134	6.672	61.39332366		3.491	6.335	81.46662847	
4	5.401	3.583	50.73960368	50.7396037	4.629	3.035	52.52059308	88.95054747	3.842	6.57	71.00468506	80.59642855	3.156	6.266	98.54245881	96.21745968
					5.479	2.431	125.3805019		3.72	7.075	90.18817204		3.422	6.635	93.89246055	
5	6.102	3.464	76.15473441	74.9090504	5.847	2.488	135.0080386	102.4076971	3.594	6.468	79.96661102	72.24656778	3.295	6.712	103.7025797	88.57287598
	6.139	3.535	73.66336634		4.848	2.855	69.80735552		4.034	6.637	64.52652454		3.581	6.211	73.4431723	
6	6.2432	3.827	63.13561536	76.4470486	5.086	2.885	76.29116118	76.05283386	4.517	7.583	67.87690945	71.78781522	3.78	6.4	69.31216931	73.57619216
	6.5998	3.478	89.75848189		7.393	4.205	75.81450654		4.222	7.418	75.69872099		4.093	7.279	77.840215	
7	6.1206	3.286	86.26293366	93.0804296					5.365	8.16	52.09692451	63.81762677	3.701	6.77	82.92353418	85.02157821
	6.0709	3.037	99.89792558						4.644	8.152	75.53832903		3.812	7.133	87.11962225	
8	6.3	3.176	98.3627204	80.7784807					4.122	6.926	68.02523047	71.09903499	3.035	5.72	88.46787479	83.34790938
	6.6877	4.098	63.19424109						4.05	7.054	74.17283951		2.641	4.707	78.22794396	
9	6.2556	3.881	61.18526153	71.6770726									3.093	5.815	88.00517297	98.34676629
	6.2994	3.458	82.16888375										2.981	6.221	108.6883596	
10	5.8493	3.735	56.60776439	66.3182855												
	5.9885	3.402	76.02880658													
11	5.747	3.274	75.53451436	83.5654402												
	5.905	3.082	91.596366													
12	6.298	2.921	115.6110921	113.070414												
	6.478	3.077	110.5297368													
