# Supplementary material for: Carbohydrate sulfotransferase 14 gene deletion induces dermatan sulfate deficiency and affects collagen structure and bowel contraction
Source: PLoS One. 2025 May 6;20(5):e0320943. doi: 10.1371/journal.pone.0320943 (PMC12054877; doi:10.1371/journal.pone.0320943)
Supplement: S5 Table — (PDF) [file pone.0320943.s011.pdf]

	No.	1						2						3						4					
		mucosa	*muscularis mucosa*	*submucosa*	*total muscle*	*circular muscle*	*longitudinal muscle*	*mucosa*	*muscularis mucosa*	*submucosa*	*total muscle*	*circular muscle*	*longitudinal muscle*	*mucosa*	*muscularis mucosa*	*submucosa*	*total muscle*	*circular muscle*	*longitudinal muscle*	*mucosa*	*muscularis mucosa*	*submucosa*	*total muscle*	*circular muscle*	*longitudinal muscle*
	1	225.388	31.105	8.27	255.663	159.373	112.654	87.903	33.243	11.843	268.788	131.431	122.478	199.966	41.454	11.956	140.639	103.226	43.126	93.373	22.804	13.379	216.259	141.46	52.306
	2	242.997	26.838	6.346	289.814	155.587	102.258	154.651	31.056	26.669	304.821	185.026	120.497	97.127	29.507	10.359	157.654	93.103	43.892	237.5	18.467	11.453	211.691	148.074	53.775
	3	250.307	38.754	6.194	244.134	153.727	90.321	155.043	46.379	26.319	280.836	199.293	100.774	123.738	28.216	20.043	139.67	111.452	45.312	83.376	21.73	12.422	222.606	137.038	66.397
	4	255.595	42.815	15.258	225.863	144.08	89.31	86.258	42.236	17.396	271.166	192.466	94.606	192.403	21.521	19.78	182.263	161.716	44.44	141.699	39.478	8.695	164.597	146.86	78.888
	5	149.25	26.531	6.783	231.897	142.519	83.986	148.544	32.056	15.984	296.02	192.847	91.925	266.145	21.178	13.707	213.297	169.827	39.172	107.865	20.487	13.437	205.059	154.079	59.263
	6	160.567	26.925	9.223	254.198	120.162	116.684	89.932	29.652	15.46	287.609	200.304	98.771	164.56	26.351	23.7	280.332	244.644	40.168	250.188	24.751	10.686	231.388	157.232	72.219
	7	134.731	32.415	10.45	261.359	137.493	119.771	117.616	30.33	19.685	282.367	197.425	74.575	144.189	18.75	30.134	277.205	208.631	45.746	123.349	23.894	9.702	211.763	145.36	45.979
	8	232.311	35.139	10.712	263.121	142.73	120.754	107.668	26.944	9.972	244.667	219.514	65.78	222.668	28.392	29.438	221.741	164.132	35.351	151.173	44.529	11.785	192.831	132.026	59.833
	9	146.156	35.892	11.577	239.556	140.379	128.465	161.482	24.525	9.938	290.853	211.767	54.233	255.089	25.632	24.17	204.673	160.286	26.48	206.147	49.967	8.784	191.559	132.847	55.512
	10	156.664	26.904	9.176	230.162	151.223	103.765	214.876	34.785	10.766	281.67	192.526	74.71	162.842	18.262	39.295	222.54	176.124	47.258	67.492	28.327	20.487	208.751	141.378	52.188
	11	219.051	30.291	7.371	213.82	140.412	95.314	145.967	26.723	19.815	188.181	181.358	70.818	177.135	18.557	16.816	203.042	161.984	44.265	221.664	23.991	21.154	195.387	146.233	44.789
	12	99.56	20.009	9.138	210.569	149.25	82.69	105.613	32.141	15.471	198.462	187.725	81.317	167.009	21.178	13.987	206.245	163.768	37.618	144.919	24.119	10.613	190.61	140.282	65.19
	13	93.575	24.125	9.727	252.864	152.468	52.607	113.555	27.641	23.177	188.926	186.339	91.665	155.601	21.581	10.581	200.76	162.761	46.058	177.858	37.596	11.453	180.57	145.599	62.864
	14	159.553	22.114	6.588	287.609	142.574	63.543	252.1	25.849	16.779	232.381	211.782	89.173	192.141	25.12	13.594	217.981	152.154	35.564	176.464	38.828	11.719	180.544	154.355	55.163
Chst14 $^{+/+}$	15	153.561	16.25	8.75	231.314	143.486	80.505	139.089	23.541	16.982	236.106	209.529	62.902	189.971	40.268	19.174	192.065	158.308	34.882	210.143	30.554	12.422	185.356	156.996	62.309
	16	196.363	16.262	7.927	241.514	166.524	68.25	149.365	24.934	18.262	231.955	180.548	67.242	188.164	27.474	20.245	189.859	169.588	42.987	89.732	14.958	16.149	172.274	148.094	57.906
	17	242.109	19.253	12.09	181.622	185.266	74.292	183.25	26.358	21.695	255.912	139.689	59.055	176.803	12.337	16.29	195.578	158.308	34.525	130.527	25.122	14.907	171.119	143.936	60.5
	18	183.651	30.233	10.425	232.181	164.129	89.375	191.746	31.916	14.404	230.671	115.153	58.477	184.79	14.617	15.597	194.479	142.112	42.039	101.375	27.244	22.36	189.88	138.059	53.357
	19	119.191	16.875	10.416	239.819	162.208	61.032	167.903	27.039	11.505	276.014	99.379	86.992	171.052	22.788	8.802	193.064	136.436	35.906	278.103	33.448	9.938	196.037	129.214	45.979
	20	107.225	24.574	11.219	224.948	186.331	31.03	111.498	32.84	15.543	224.93	105.59	74.42	161.658	24.552	18.125	175.775	135.532	46.012	189.477	22.117	11.18	185.323	121.261	47.998
	21	232.04	16.226	13.34	229.513	145.67	78.744	156.752	28.16	18.449	185.858	126.811	76.051	193.827	19.573	13.364	152.357	152.427	45.906	213.379	23.438	11.785	192.459	125.728	36.174
	22	160.859	23.758	11.008	242.332	152.375	73.891	139.133	27.448	30.742	200.004	133.793	68.323	147.167	13.693	12.228	143.824	142.536	55.015	68.333	33.792	5.555	199.068	134.458	28.381
	23	182.549	14.59	12.672	238.233	171.648	65.388	114.262	34.684	39.911	247.906	122.277	81.012	146.793	21.178	14.315	158.118	123.738	51.449	152.509	34.871	10.613	203.256	141.814	38.025
	24	205.921	17.331	13.287	233.081	163.651	68.066	181.543	34.684	19.409	219.141	125.564	63.11	165.789	23.626	18.093	157.713	114.747	46.497	133.681	33.425	6.211	211.176	140.771	37.555
	25	187.635	17.677	11.931	244.639	160.622	55.656	154.093	56.444	8.616	212.233	138.089	80.17	206.391	13.917	9.841	158.253	117.384	45.063	180.408	17.568	8.784	218.506	134.481	35.069
	26	206.987	22.017	16.635	234.826	154.169	85.303	96.334	60.803	12.843	240.571	140.57	72.7	201.966	14.163	6.161	165.436	123.044	36.473	158.935	17.435	11.453	220.296	145.768	38.907
	27	230.118	20.155	16.593	288.918	159.617	66.909	92.428	41.779	12.058	284.331	159.752	68.908	122.884	24.294	7.699	175.083	114.828	35.477	176.883	18.962	10.015	199.01	159.124	38.569
	28	180.285	19.862	11.733	306.872	164.529	61.217	143.26	63.376	23.203	284.483	161.285	64.847	161.845	19.986	9.662	171.893	113.612	35.209	170.889	23.569	8.333	196.772	143.049	45.217
	29	189.053	26.257	10.68	265.351	158.925	74.376	90.502	44.745	15.371	266.111	143.955	67.23	179.309	17.432	12.228	150.505	120.31	41.14	99.455	30.68	10.244	188.559	186.104	34.782
	30	189.933	27.457	8.549	262.918	166.328	70.28	199.165	30.726	20.708	277.219	120.343	105.59	264.211	20.166	8.091	147.584	114.766	45.771	183.6	28.598	11.785	182.233	155.495	49.72
	average	183.106167	24.95446667	10.4689333	245.2903333	154.5818333	82.21453333	141.7177	34.43456667	17.9658333	249.673067	163.7376667	79.6117	179.4411	22.52543333	16.2491667	186.320933	145.7161333	41.6267	157.349867	27.82496667	11.9167667	197.164633	144.2391667	51.16046667

	No.	5						6						7						8					
		mucosa	*muscularis mucosa*	*submucosa*	*total muscle*	*circular muscle*	*longitudinal muscle*	*mucosa*	*muscularis mucosa*	*submucosa*	*total muscle*	*circular muscle*	*longitudinal muscle*	*mucosa*	*muscularis mucosa*	*submucosa*	*total muscle*	*circular muscle*	*longitudinal muscle*	*mucosa*	*muscularis mucosa*	*submucosa*	*total muscle*	*circular muscle*	*longitudinal muscle*
	1	202.415	22.028	8.967	139.676	110.989	44.864	119.465	22.541	18.961	141.684	87.59	33.344	202.752	24.547	12.989	118.516	86.747	40.435	228.427	73.805	15.16	234.377	101.249	104.502
	2	121.609	22.186	5.785	160.24	117.243	48.877	187.142	19.76	8.907	108.587	90.939	34.033	177.463	21.424	16.222	115.339	79.636	37.583	206.472	41.888	13.932	234.933	112.681	81.636
	3	256.299	13.479	4.337	158.137	114.925	42.133	200.383	24.013	8.182	101.519	85.354	29.501	184.323	25.108	8.369	131.685	85.16	34.418	169.157	55.853	8.359	227.068	114.547	55.964
	4	117.61	24.074	6.327	162.09	115.914	41.749	100.846	24.837	10.882	119.396	91.607	22.438	207.352	22.473	9.836	142.087	106.899	25.046	127.514	23.179	10.574	214.507	129.259	66.044
	5	245.931	28.065	6.23	159.19	116.525	46.812	178.072	22.274	7.517	131.689	87.504	40.363	171.862	28.623	7.594	120.961	85.114	33.719	229.336	28.416	10.719	212.072	132.486	68.931
	6	226.641	36.807	9.732	155.083	97.142	50.498	163.824	17.429	10.203	113.049	81.038	33.5	126.177	39.191	7.594	106.761	79.512	33.896	160.363	34.891	12.273	239.281	152.575	68.626
	7	185.842	28.099	5.416	180.595	112.848	28.768	82.613	18.37	11.42	105.968	80.727	33.257	199.786	21.297	8.087	122.652	85.307	30.842	174.822	31.227	12.461	241.59	161.518	67.393
	8	176.779	21.22	12.074	156.51	122.859	55.957	200.063	17.251	12.269	105.871	99.44	21.378	214.613	28.919	9.331	115.222	78.799	32.782	214.651	33.483	9.49	212.891	128.173	125.511
	9	151.792	16.247	10.384	132.012	107.946	61.278	159.53	14.321	12.348	117.68	96.615	31.751	200.123	27.744	14.308	107.282	74.963	39.191	179.195	30.777	10.276	205.607	133.583	59.722
	10	79.626	23.847	25.504	147.61	101.702	40.426	170.818	19.534	12.222	122.417	89.804	22.473	157.487	31.866	10.575	113.312	74.414	40.435	142.597	26.168	9.49	193.728	136.105	90.426
	11	230.058	36.292	18.034	149.076	117.783	43.818	129.087	18.662	14.308	115.976	87.165	23.9	138.87	28.972	4.977	95.285	67.833	36.976	185.293	32.423	10.046	200.855	118.72	94.835
	12	173.157	22.645	13.932	135.203	103.377	42.755	179.823	13.429	15.059	118.431	98.238	26.626	167.027	16.435	9.248	93.745	80.931	38.474	260.4	39.582	7.58	202.947	128.928	87.494
	13	170.898	18.165	14.147	134.239	100.468	28.721	148.728	17.705	19.375	130.976	99.9	17.206	154.804	17.617	10.05	110.081	82.379	32.616	231.258	36.137	10.647	275.468	133.978	70.159
	14	189.177	16.496	12.16	122.741	97.914	37.569	198.193	20.311	8.087	133.597	86.543	18.04	212.29	16.739	12.079	116.879	88.524	25.513	157.794	31.45	10.574	172.899	113.478	68.434
Chst14 $^{+/+}$	15	153.893	20.218	10.614	121.373	81.451	32.524	158.498	18.38	8.907	104.428	78.749	20.595	119.169	30.936	9.331	122.721	102.772	35.823	191.408	21.762	16.247	225.765	139.079	49.422
	16	177.914	13.679	13.882	117.565	81.257	32.88	181.366	24.396	5.13	108.644	85.985	33.111	164.857	18.04	7.967	120.275	101.153	27.611	158.835	30.01	15.958	186.437	133.583	58.779
	17	222.578	24.992	8.643	122.048	93.723	23.977	129.321	19.672	6.158	112.597	86.221	36.724	159.586	20.062	15.552	135.273	98.378	30.022	150.483	33.25	12.273	196.825	112.246	69.748
	18	206.338	14.545	13.764	126.123	85.705	26.287	126.959	19.042	9.836	122.314	71.645	30.729	156.271	36.892	14.199	129.405	95.506	23.351	193.919	32.898	10.046	180.612	134.036	79.487
	19	98.099	12.273	16.199	126.496	92.793	31.586	188.891	25.717	15.301	122.071	72.228	26.488	137.953	16.186	9.185	119.052	82.905	29.632	197.451	46.307	11.284	216.076	139.569	101.663
	20	185.583	18.587	12.571	121.871	91.019	27.506	206.084	23.118	10.26	114.275	70.767	25.007	136.378	23.318	11.471	97.372	75.741	32.186	135.637	41.272	6.71	199.952	163.282	94.655
	21	194.104	24.331	10.003	117.458	84.682	35.079	174.489	13.91	4.859	126.321	79.726	22.404	197.871	30.634	9.657	108.881	65.818	28.945	200.975	42.441	7.477	216.396	140.362	117.028
	22	88.475	26.566	14.129	113.943	99.364	38.649	172.741	13.91	8.346	142.163	82.258	33.644	188.795	29.501	26.757	94.761	72.379	32.563	173.328	48.614	11.215	173.065	106.918	104.346
	23	206.162	20.371	13.832	139.948	110.231	39.029	112.147	16.281	4.486	162.416	110.557	32.108	167.599	38.834	32.97	102.992	61.771	26.597	121.496	41.103	8.359	214.015	135.642	75.798
	24	138.152	16.776	13.713	143.842	91.489	31.152	186.728	16.222	5.351	138.558	132.986	24.197	137.058	22.43	37.335	104.408	77.421	32.33	150.05	26.287	7.979	244.697	139.569	90.246
	25	167.523	19.801	6.354	134.995	107.101	30.479	151.971	14.982	5.015	123.307	102.146	29.244	160.811	16.364	23.118	103.507	71.16	30.431	171.7	39.817	13.246	250.86	123.54	76.511
	26	131.038	17.556	6.913	147.243	112.87	24.687	108.692	11.966	13.182	126.723	89.83	16.329	189.862	25.717	16.435	105.514	69.773	35.584	229.969	36.479	7.979	225.404	135.877	83.126
	27	168.701	18.949	5.07	146.385	120.92	40.152	180.854	26.429	12.269	146.895	105.75	34.356	141.503	22.091	25.649	98.049	70.108	26.713	154.943	30.777	7.049	219.403	122.524	71.42
	28	192.824	11.756	6.175	188.085	129.552	34.375	160.556	23.143	22.395	137.006	105.174	32.551	150.345	18.807	24.293	103.08	72.89	34.803	151.482	23.511	8.359	175.771	140.567	74.371
	29	190.616	12.845	11.06	158.652	131.014	28.768	176.396	13.429	15.136	152.014	104.335	34.22	137.493	24.984	16.364	121.4	75.664	42.689	110.22	31.721	8.723	221.796	132.093	82.366
	30	158.027	16.39	6.502	173.142	126.055	27.527	202.523	12.222	12.596	129.957	106.441	32.33	131.648	20.538	17.418	116.554	78.187	38.373	155.521	29.171	13.246	243.845	117.452	101.548
	average	173.595367	20.64283333	10.7484333	143.0523667	105.8953667	37.29606667	161.226767	18.7752	10.9655667	124.550967	91.5754	28.3949	166.404267	24.8763	14.632	113.1017	80.92813333	32.98596667	177.156533	35.8233	10.5910333	215.304733	130.4539667	81.3397

	No.	1						2						3						4					
		mucosa	*muscularis mucosa*	*submucosa*	*total muscle*	*circular muscle*	*longitudinal muscle*	*mucosa*	*muscularis mucosa*	*submucosa*	*total muscle*	*circular muscle*	*longitudinal muscle*	*mucosa*	*muscularis mucosa*	*submucosa*	*total muscle*	*circular muscle*	*longitudinal muscle*	*mucosa*	*muscularis mucosa*	*submucosa*	*total muscle*	*circular muscle*	*longitudinal muscle*
	1	195.109	19.524	13.812	166.324	114.898	67.986	194.144	28.768	14.953	176.141	127.078	43.897	285.554	49.143	32.607	119.28	59.979	43.265	225.295	25	8.385	196.349	184.204	61.046
	2	117.244	17.206	15.75	114.128	100.933	75.69	185.736	19.465	15.159	152.437	107.663	42.367	172.778	41.758	17.786	104.377	58.477	44.235	202.797	28.858	10.308	224.902	233.452	52.974
	3	119.616	14.787	13.643	123.63	71.528	59.473	186.873	26.668	13.246	138.177	98.828	47.352	231.686	24.469	14.164	117.672	63.104	31.353	123.25	41.552	17.002	169.742	164.81	57.35
	4	165.507	18.083	14.321	137.338	84.211	59.629	72.338	30.776	23.179	127.255	91.476	47.417	251.686	23.635	13.665	96.479	62.124	39.771	196.254	31.35	12.5	162.62	193.847	53.224
	5	155.176	28.818	13.77	137.245	84.925	55.766	198.381	31.152	15.413	138.877	85.51	46.257	131.489	26.322	13.721	143.541	83.784	46.778	196.154	32.5	11.25	164.022	160.998	64.578
	6	158.707	22.95	22.891	159.778	111.359	71.255	95.227	20.739	21.184	113.148	88.482	44.039	273.08	32.607	10.686	148.97	76.16	45.811	73.24	29.712	11.319	184.535	137.027	55.481
	7	146.209	14.076	16.329	157.504	108.8	68.429	207.265	33.483	16.39	112.322	88.718	57.063	144.228	29.865	11.249	166.445	67.313	45.676	171.801	20.691	5.59	202.561	120.266	52.351
	8	183.151	13.123	20.062	149.87	103.187	62.481	199.703	14.532	16.949	113.771	80.996	33.852	224.978	24.845	20.563	125.521	70.943	52.189	68.886	23.881	9.52	217.676	114.086	51.675
	9	142.342	12.457	13.77	159.207	111.255	65.185	170.091	20.285	15.005	132.462	86.449	30.01	126.598	29.632	23.992	135.284	71.342	64.787	171.796	28.339	8.004	193.427	112.284	51.539
	10	154.588	18.137	9.836	188.117	139.282	64.047	119.034	33.645	12.336	124.84	89.233	36.649	217.618	20.374	22.395	139.888	60.373	43.955	185.27	22.638	8.004	181.663	138.253	49.497
	11	167.433	22.644	8.972	181.306	131.975	66.769	216.538	23.643	6.23	132.585	103.126	35.09	84.7	36.683	32.058	145.108	81.657	53.647	161.56	43.175	18.583	202.954	145.473	34.483
	12	166.473	10.127	8.461	189.743	137.873	68.429	140.716	22.43	5.287	147.614	115.087	40.186	200.766	20.563	27.582	152.025	79.006	60.143	84.724	52.157	19.405	205.034	121.256	58.269
	13	136.752	11.885	8.369	173.565	111.734	43.988	203.862	17.445	6.71	145.517	93.391	43.827	204.852	16.713	23.863	151.964	94.724	35.158	198.891	36.912	16.25	204.347	91.464	72.252
	14	224.338	24.229	9.717	179.112	124.742	50.415	205.848	23.937	10.719	130.293	87.945	39.029	115.421	21.372	15.811	146.104	86.712	67.368	148.503	32.137	10	213.867	95	71.981
Chst14 $^{-/-}$	15	118.452	25.513	10.031	181.047	112.363	39.833	132.216	19.345	9.072	125.008	92.211	35.943	243.053	17.436	11.249	142.142	95.936	57.264	100.755	29.182	5.59	193.459	102.546	82.84
	16	157.662	20.865	8.798	184.65	118.98	41.73	185.773	24.734	7.477	120.929	84.606	35.289	181.251	15.565	24.845	134.219	83.355	52.189	168.272	27.5	9.52	212.161	121.353	57.554
	17	203.442	17.429	13.313	194.225	112.237	52.815	164.23	17.356	10.046	113.141	90.34	45.153	119.106	25.609	31.279	126.275	87.072	67.368	145.484	20.039	9.1	247.541	152.612	70
	18	137.267	18.807	7.795	187.891	112.942	51.298	227.368	16.199	15.057	124.523	89.207	44.738	277.961	18.798	42.527	127.805	89.807	59.055	163.502	21.83	16.817	291.874	167.518	80.039
	19	148.865	12.989	8.346	188.491	116.442	57.944	157.763	32.992	15.057	140.682	115.209	38.123	116.796	27.244	44.112	134.448	78.095	54.884	169.198	23.585	14.577	260.012	178.82	58.162
	20	186.306	17.329	9.516	162.802	119.913	51.011	216.223	20.093	8.986	145.372	108.382	41.291	262.194	20.222	31.938	138.134	73.421	50.444	84.945	23.881	13.05	222.531	163.234	38.931
	21	201.279	10.575	8.182	178.078	102.235	67.334	142.405	22.464	6.71	138.474	93.091	37.383	148.612	18.467	22.012	140.833	84.823	49.143	169.779	26.516	15.207	228.326	149.838	46.921
	22	157.22	20.463	7.465	186.857	103.776	51.853	179.04	24.482	12.461	166.996	106.445	36.158	194.46	22.395	13.889	130.168	91.66	51.113	86.756	20.691	12.748	218.66	146.783	34.118
	23	90.604	16.796	7.695	196.865	105.616	47.032	184.499	13.707	8.723	174.187	120.672	33.852	204.49	28.679	13.437	121.84	92.648	55.401	161.245	22.638	15.207	189.159	150.005	36.443
	24	138.774	15.564	10.428	205.449	124.123	53.252	157.842	14.208	15.159	168.242	109.124	38.225	202.754	26.205	14.958	125.472	84.554	52.82	153.851	26.369	7.5	164.64	154.444	36.785
	25	131.918	16.922	9.717	201.788	103.322	59.356	132.169	19.345	11.284	158.847	102.279	31.769	185.696	30.327	13.205	126.201	89.805	56.655	92.077	17.545	10.308	192.293	143.962	32.716
	26	109.217	18.991	6.613	179.169	125.014	60.056	170.169	19.021	15.005	156.915	109.153	45.153	187.984	27.273	18.087	129.586	78.786	55.136	200.285	11.18	7.906	222.963	157.371	42.5
	27	193.746	16.692	8.346	164.163	140.59	72.204	111.684	22.909	19.703	190.865	126.342	37.673	244.294	21.444	15.565	118.742	88.131	52.777	161.293	26.516	10.308	189.819	143.668	44.878
	28	64.879	13.313	11.023	186.281	116.374	69.506	185.715	20.093	17.445	132.116	94.383	40.57	101.293	17.786	12.969	113.37	76.742	45.15	110.807	28.339	10.753	213.237	143.723	39.941
	29	135.615	11.128	6.495	205.058	105.726	63.355	161.979	28.196	11.284	138.34	112.839	35.289	160.421	34.448	3.727	118.247	66.764	54.658	136.301	19.08	6.25	188.481	155.694	38.588
	30	176.043	14.429	12.825	153.431	122.953	64.888	113.238	14.585	7.266	152.697	87.52	51.589	222.53	26.759	6.334	104.288	73.975	47.025	143.75	48.541	10.607	271.71	207.534	45.962
	average	152.7978	17.19503333	11.2097	172.4370667	112.6436	59.43363333	167.268967	22.55656667	12.7831667	141.092433	99.52616667	40.50776667	190.610967	25.88793333	20.0091667	130.814267	78.37573333	51.17393333	148.557367	28.0778	11.3856	207.6855	148.3841667	52.43593333

	No.	5						6						7					
		mucosa	*muscularis mucosa*	*submucosa*	*total muscle*	*circular muscle*	*longitudinal muscle*	*mucosa*	*muscularis mucosa*	*submucosa*	*total muscle*	*circular muscle*	*longitudinal muscle*	*mucosa*	*muscularis mucosa*	*submucosa*	*total muscle*	*circular muscle*	*longitudinal muscle*
	1	174.923	26.631	12.77	243.303	158.953	97.63	204.15	35.125	14.987	220.68	169.926	83.202	184.059	26.492	17.445	134.947	94.711	54.101
	2	108.199	32.2	12.278	277.965	184.005	97.67	169.885	18.605	17.275	248.618	177.624	69.91	74.15	24.195	12.523	153.325	111.822	64.472
	3	88.489	21.122	12.403	204.173	166.645	70.206	149.043	20.531	8.682	248.138	170.951	72.172	165.036	40.127	11.215	138.518	101.448	45.954
	4	132.962	19.255	16.314	215.757	196.157	54.035	61.015	20.643	19.255	243.293	149.11	71.906	145.19	42.877	14.585	183.243	94.284	53.131
	5	130.238	36.647	9.03	255.395	202.151	64.496	191.927	22.36	13.867	227.602	146.99	70.162	175.703	41.888	18.524	164.144	94.613	41.497
	6	110.638	29.819	11.163	230.424	245.96	49.674	162.513	19.999	18.892	252.858	144.244	62.028	182.543	38.225	15.613	184.173	85.036	59.448
	7	178.096	34.817	10	264.485	190.621	78.149	144.84	27.315	9.446	227.896	136.996	60.889	70.49	18.524	12.461	155.117	98.536	76.45
	8	88.689	34.551	8.682	278.451	140.899	64.687	150.614	24.082	11.163	214.171	135.789	83.387	193.558	21.762	13.932	141.556	113.879	57.806
	9	130.256	21.122	8.944	268.036	136.957	103.065	183.653	15.342	7.942	204.954	143.629	73.681	168.647	40.359	16.199	124.609	114.152	33.737
	10	156.54	30.28	8.77	262.207	132.498	99.294	159.865	23.598	10.669	176.11	159.619	77.109	163.499	24.066	18.524	158.651	128.945	41.59
	11	127.577	23.696	10	316.181	158.473	106.059	45.487	11.435	12.949	178.224	166.686	91.076	163.888	44.616	7.58	157.368	109.316	45.954
	12	202.125	32.176	6.679	319.268	190.152	100.74	131.191	23.858	8.77	186.137	159.272	80.858	162.475	24.922	12.461	149.058	100.687	55.518
	13	164.195	32.839	8.32	337.831	206.026	86.689	128.136	17.408	11.163	194.218	168.973	72.799	183.328	34.979	15.413	121.646	95.308	33.529
	14	150.405	24.806	6.202	304.429	206.149	67.855	73.754	27.539	8.77	226.826	155.217	56.034	85.173	34.012	8.811	136.075	90.064	43.256
Chst14 $^{-/-}$	15	98.486	30.809	5.262	373.681	181.966	90.543	145.116	25.114	8.77	233.734	155.711	32.462	185.031	19.702	1.246	138.58	82.252	32.494
	16	155.87	30.28	9.687	355.709	162.499	92.791	172.937	21.085	13.185	235.818	179.244	62.755	191.999	19.021	21.798	141.551	80.919	39.029
	17	85.086	32.956	7.844	256.144	162.523	100.457	108.766	23.402	11.094	214.196	182.021	63.849	98.512	26.755	19.702	129.743	83.302	55.054
	18	117.201	53.85	7.232	185.01	155.336	117.26	113.14	33.854	17.628	233.032	214.329	70.795	201.667	19.504	12.273	140.118	72.659	50.846
	19	135.671	25.898	12.465	241.15	137.243	121.588	156.5	25.084	17.231	252.59	189.198	64.924	174.6	22.43	21.184	141.161	75.314	49.859
	20	156.893	25.54	9.922	221.321	141.079	121.557	152.603	16.64	11.435	261.457	209.681	67.137	143.013	21.762	12.523	127.869	81.987	67.795
	21	83.847	22.803	4.961	241.844	134.16	112.97	131.472	21.661	12.403	282.509	200.339	74.004	138.765	26.168	22.291	130.786	90.03	75.694
	22	126.809	22.87	7.545	236.18	131.969	96.943	148.474	19.255	8.682	272.768	212.675	80.496	134.636	25.077	19.504	123.369	86.233	50.293
	23	187.877	19.845	9.687	254.266	177.117	97.425	151.017	17.408	13.867	273.253	223.231	68.89	147.45	35.267	10.046	127.765	91.169	47.58
	24	135.671	21.122	6.202	264.698	155.929	119.386	192.283	18.059	10.597	321.342	221.917	62.028	202.332	39.66	6.23	138.63	93.49	39.894
	25	92.833	30.203	5.262	248.093	148.609	124.186	169.382	16.314	17.758	322.882	178.853	79.968	124.914	24.922	16.949	143.814	91.382	50.831
	26	142.052	37.209	7.232	243.556	159.345	111.552	146.361	21.194	10.228	346.753	172.206	86.047	97.235	25.077	10.276	139.879	92.622	38.709
	27	143.924	26.544	9.03	242.977	165.65	129.004	118.402	23.858	9.446	258.329	179.759	108.482	167.274	31.45	9.49	142.975	78.859	44.738
	28	126.061	32.272	9.922	264.686	181.679	107.514	129.806	32.557	10.524	306.445	173.323	70.184	137.635	23.279	14.208	146.679	92.245	36.287
	29	146.088	37.002	8.32	242	215.946	129.087	155.202	21.978	9.687	276.866	147.241	124.883	148.62	24.953	7.881	151.824	91.407	39.128
	30	128.765	31.23	11.701	288.275	222.074	141.922	134.274	22.36	5.547	170.856	140.423	106.522	60.188	39.128	11.756	141.704	99.602	34.465
	average	133.548867	29.34646667	9.12763333	264.5831667	171.6256667	98.48113333	142.726933	22.25543333	12.0637333	243.751833	172.1725667	74.95463333	149.053667	29.3733	13.7547667	143.629233	93.87576667	48.63796667
