# Supplementary material for: Carbohydrate sulfotransferase 14 gene deletion induces dermatan sulfate deficiency and affects collagen structure and bowel contraction
Source: PLoS One. 2025 May 6;20(5):e0320943. doi: 10.1371/journal.pone.0320943 (PMC12054877; doi:10.1371/journal.pone.0320943)
Supplement: S6 Table — (PDF) [file pone.0320943.s012.pdf]

	Chst14+/+															Chst14-/-														
	1					2					3					1					2					3				
	1	2	3	4	5	1	2	3	4	5	1	2	3	4	5	1	2	3	4	5	1	2	3	4	5	1	2	3	4	5
1	43.265	39.598	42.449	43.542	42.457	40.824	50.295	40.824	46.531	43.265	41.665	34.286	40.816	34.441	35.102	40.816	42.457	37.56	27.803	28.196	35.112	39.192	32.745	30.204	40	30.204	45.692	32.663	46.559	34.295
2	47.375	35.928	36.744	35.928	45.714	40.033	46.178	44.898	31.02	36.14	49.903	31.931	37.551	34.286	35.918	41.641	37.56	34.295	24.975	37.024	36.744	40.816	36.735	37.551	40.816	37.631	35.863	31.879	44.089	38.402
3	46.538	43.111	34.286	31.063	47.354	40.89	35.918	37.56	42.449	43.396	48.274	36.744	35.918	33.509	45.743	41.705	44.898	29.399	30.204	36.066	39.192	38.367	33.469	29.433	39.218	33.469	38.885	39.184	38.367	37.551
4	43.265	41.705	40.816	45.78	31.847	41.633	44.928	32.663	25.319	44.36	40.947	30.204	36.744	33.469	40.53	38.402	42.48	35.112	23.118	29.263	36.735	45.714	39.184	38.445	45.78	37.56	42.457	34.286	47.375	36.744
5	40.816	46.538	40.816	40.133	37.551	39.218	48.225	43.296	41.633	37.176	41.02	33.469	35.928	34.286	41.085	34.295	39.184	31.02	30.215	42.363	31.847	41.633	44.082	30.204	37.56	34.295	31.753	33.469	34.286	44.353
6	44.082	38.376	36.002	34.441	38.367	45.78	47.375	39.192	42.48	41.182	46.595	41.641	41.633	31.837	37.462	42.449	31.031	40.824	26.122	34.266	32.004	43.227	39.184	32.663	38.402	29.949	36.771	34.286	31.7	34.286
7	40.816	36.744	42.457	36.771	40	48.17	50.612	39.192	38.376	38.584	53.287	44.965	41.633	38.402	36.671	35.928	37.631	25.62	25.306	34.295	33.865	38.506	44.905	41.641	42.457	40	38.376	34.295	35.918	34.286
8	37.586	37.631	43.265	37.631	36.744	44.112	34.295	36.553	51.032	49.528	49.088	40.008	35.102	35.928	44.905	44.898	40.008	31.837	29.399	32.51	35.807	32.653	37.586	40	30.204	32.663	39.192	31.879	40.849	40.208
9	40.816	40.947	39.184	31.02	43.265	44.965	40.824	37.302	49.149	43.755	40.89	38.679	39.184	37.693	43.885	45.714	34.325	34.101	26.122	34.94	30.016	42.078	36.37	44.898	48.191	32.5	35.928	35.928	39.252	39.218
10	47.523	43.296	32.663	31.063	37.56	32.653	40.824	45.692	49.007	38.791	34.528	28.676	34.295	37.23	29.399	37.56	27.803	35.187	24.49	36.37	38.988	34.11	38.445	32.653	42.449	27.441	36.916	34.295	44.082	34.286
11	45.164	38.919	48.17	38.367	36.771	38.506	40.816	40.008	42.449	37.772	47.6	46.531	40.008	40.849	31.245	42.449	31.847	29.308	25.306	43.265	31.245	39.866	40.824	40.008	37.586	33.479	37.462	23.8	43.273	40.133
12	40.849	32.53	37.551	42.731	47.107	40.008	40.008	44.15	49.823	44.27	42.832	35.918	35.918	49.655	44.353	37.56	35.102	36.251	26.939	38.506	36.002	45.831	35.928	31.02	40.816	30.599	40.324	37.586	47.389	37.56
13	42.52	39.192	40.033	49.007	40.816	38.376	40.849	43.335	45.722	56.616	37.56	44.965	34.286	30.479	41.472	40.033	35.928	42.425	25.306	36.15	33.479	37.239	40.849	30.204	37.56	35.918	33.028	37.551	39.201	31.847
14	43.961	41.303	40.816	40.816	46.531	37.56	53.567	40.947	40.033	43.388	51.032	34.295	31.847	34.873	28.571	35.928	39.192	37.462	27.755	33.059	33.469	43.388	27.755	48.98	33.469	40	34.061	35.955	48.707	44.089
15	42.902	43.519	43.265	39.489	42.457	40.008	36.002	38.367	49.823	38.237	39.218	36.735	36.735	37.453	36.066	34.286	32.663	37.132	26.122	26.376	34.6075	41.832	42.52	33.479	35.112	34.295	34.873	31.837	39.218	32.097
16	49.149	35.918	36.771	39.184	42.023	42.457	30.215	44.112	35.102	39.598	50.717	35.14	35.928	35.891	50.75	39.192	35.102	28.571	26.135	43.265		39.09	40.033	40.816	44.905	37.816	47.354	33.479	37.869	33.469
17	44.112	39.192	35.064	38.376	40.759	44.15	42.48	41.832	40.824	45.831	44.928	40.816	33.559	30.38	43.227	43.273	36.816	24.009	27.755	33.865		44.898	36.002	32.653	38.679	35.102	38.445	34.441	44.353	36.771
18	42.77	38.376	38.945	36.744	39.758	38.402	29.388	50.691	35.102	39.319	47.41	50.639	29.388	37.693	36.744	36.735	35.928	23.8	23.688	39.218		35.918	35.955	32.653	37.382	37.631	40.816	32.694	40.025	31.837
19	41.02	38.402	38.988	42.48	35.055	39.26	35.102	37.869	44.112	39.319	39.184	40.816	33.479	33.954	42.449	40.008	41.665	26.852	28.583	38.885		42.023	31.117	31.02	40.033	36.744	39.192	44.965	35.928	39.875
20	38.584	39.184	45.743	32.004	35.14	45.722	34.295	42.48	41.641	43.227	47.41	39.184	27.767	27.767	34.295	42.449	44.898	26.173	40.824	32.816		39.26	27.767	36.735	44.66	38.367	44.089	35.102	43.273	39.319
21	40.89	40.947	46.645	35.918	30.977	38.376	40.824	45.275	35.918	42.1877	33.509	40.008	32.663	37.586	36.961	40.816	28.571	32.294	30.248	28.571		37.772	33.559	33.35	32.907	33.469	42.457	35.14	42.59	38.808
22	36.744	45.714	45.495	40.008	37.737	43.265	33.628	39.808	34.295		38.376	40	30.215	33.35	39.269	36.735	37.551	30.038	27.755	30.248		35.187	33.509	35.918	34.759	31.763	42.48	37.631	39.598	43.457
23	52.404	35.112	40.816	39.192	34.94	44.905	51.429	31.405	39.26		43.335	48.98	36.771	40.669	34.101	35.112	35.918	27.767	32.663	25.943		44.082	28.583	36.15	39.933	33.559	35.112	31.02	40.324	33.509
24	44.898	34.873	40	41.641	41.816	45.722	38.376	34.101	47.523		47.354	45.78	46.595	37.56	38.722	34.286	40.947	24.175	31.847	34.451		43.265	39.26	36.735	33.469	37.693	35.918	31.837	35.14	31.837
25	41.761	34.759	38.402	38.445	41.02	40	35.112	31.847	40.849		31.063	42.449	42.48	38.402	42.52	41.641	41.705	31.837	24.828	34.579625		31.02	39.319	38.376	36.15	34.325	44.089	37.56	33.479	41.633
26	35.102	33.559	33.973	39.26	40.595	43.296	32.694	34.286	34.286		41.761	45.743	44.089	28.988	32.077	49.041	33.479	30.642	26.988			35.918	34.295	44.082	37.746	35.187	38.376	35.928	39.933	32.663
27	34.325	36.735	38.679	32.907	44.965	45.714	45.743	45.194	35.955		45.743	48.163	42.457	41.633	38.597	42.52	39.192	31.063	31.837			42.48	43.08	35.928	38.782	39.184	39.26	30.248	43.265	37.586
28	40.008	33.479	40.133	31.542	42.48	43.265	45.083	35.891	40.133		45.722	53.061	40.824	48.501		39.192	39.184	35.102	31.117			35.955	30.248	27.755	31.245	39.192	47.354	36.771	38.584	34.061
29	43.273	35.955	41.633	41.085	43.273	44.905	39.26	38.376	45.722		44.928	37.551	30.215	36.816		48.336	38.367	35.14	27.767			43.265	39.022	39.758	38.782	31.117	44.898	34.295	43.388	34.653
30	39.319	40	40.669	32.694	40.816	43.296	40.824	29.433	46.531		43.296	40	29.388	36.002		40.849	34.373	31.7	29.58			35.928	40.628	40.158	36.232	32.907	43.273	40	39.875	44.112
31	40.033	36.735	35.918	27.755	45.743	42.457	35.955	29.433	41.4033		33.479	39.184	42.449	38.402		32.653	41.633	36.37	25.319			39.6837667	30.977	43.296	36.943	34.325	36.744	35.918	32.345	37.176
32	40.208	40.033	38.115	35.112	38.402	35.102	38.376	40.008			35.955	43.273	33.479	37.56		42.023	40	27.755	30.38				39.064	30.215	31.511	40.628	42.449	36.066	30.566	38.376
33	42.52	45.831	40.816	37.23	45.722	36.771	40.824	42.52			35.112	41.641	35.112	38.376		33.509	40	33.509	28.665				36.735	36.1556563	42.457	34.73	40.947	35.112	39.933	48.225
34	43.633	44.928	41.761	40.824	38.367	48.412	41.665	31.847			36.771	35.918	40.008	38.445		31.031	34.325	32.376	29.399				38.919		26.852	43.335	41.633	36.744	42.237	40.947
35	43.265	43.542	37.772	31.837	39.192	47.375	36.735	36.744			31.879	41.665	44.082	37.23		42.457	28.583	28.711	27.863				30.303		35.955	34.325	47.347	41.705	44.413	37.266
36	36.251	47.41	40.849	32.653	39.192	47.347	42.48	30.248			34.325	36.744	36.735	36.735		44.112	40.816	33.509	29.308				35.187		37.9714857	34.325	40.824	36.002	33.23	48.796
37	35.339	32.663	41.705	38.402	40.824	44.957	36.002	32.663			45.017	38.376	43.335	42.386		24.49	33.479	30.74	28.711				37.56			33.149	37.56	33.479	38.115	36.671
38	35.918	40.849	27.767	37.772	38.367	50.612	36.744	34.286			48.336	40.816	36.735	36.744		46.538	34.295	28.513	28.478				42.52			37.693	44.905	35.102	44.248	46.135
39	41.92	43.265	40.075	36.771	41.761	41.792	47.354	36.816			47.347	35.918	44.898	33.479		30.215	36.816	40.996	31.511				35.14			34.902	41.665	29.399	44.413	40.025
40	42.948	45.722	38.018	27.767	46.595	51.454	33.149	41.665			45.976	38.367	40.824	34.295		40	44.089	33.149	32.077				35.339			36.14	31.847	31.931	37.604	33.509
41	41.02	38.367	40.075	39.489	43.664	45.896	43.265	38.570225			32.653	36.735	33.469	30.204		48.98	35.955	31.405	31.931				38.506			34.373	37.551	35.14	36.407	41.848
42	36.88	40	37.311	35.112	45.017	41.214	40.008				43.388	40.033	36.771	35.102		48.191	39.184	35.112	36.744				38.506			28.931	42.48	35.14	40.604	33.3
43	39.26	39.192	36.066	30.215	39.396	47.389	47.375				34.373	41.705	34.286	33.559		40.849	41.641	38.713	34.441				39.218			33.469	34.286	31.031	42.173	44.965
44	37.693	46.538	41.641	37.56	26.237	38.376	34.286				35.928	40.075	36.771	40.947		35.112	42.574	36.988	40.406				26.988			30.215	43.273	38.402	34.101	32.785
45	52.651	39.184	37.586	30.248	33.479	42.645	49.041				40.824	32.663	35.928	42.52		33.479	38.376	39.564	28.954				38.367			28.571	32.663	47.375	41.975	40.849
46	45.78	44.082	38.402	34.653	33.618	41.665	43.265				37.693	33.469	49.796	42.48		42.449	36.744	27.246	32.816				44.089			35.254	40.824	35.891	32.836	45.831
47	43.335	45.743	38.808	33.509	38.402	35.918	33.469				37.56	50.619	35.14	32.653		40.849	44.898	32.4206957	31.837				40.824			33.479	51.454	38.402	39.674	39.226
48	43.273	38.376	44.928	41.633	40.008	46.538	39.184				36.15	40.816	43.273	38.402		45.831	39.192		29.67				37.56			39.808	49.007	29.49	33.23	31.542
49	50.717	40.816	41.92	40.158	39.218	37.551	33.079				41.633	39.9040417	42.52	45.743		36.735	37.6751042		28.337				38.584			36.26	32.653	36.251	35.112	44.202
50	38.791	22.857	38.376	43.296	36.771	40.849	36.15				36.735		35.928	33.628		39.184			39.497				45.722			36.961	37.586	39.218	34.759	46.531
51	51.59	47.347	37.586	44.089	27.755	40.008	39.218				31.02		36.735	42.52		48.163			34.873				37.06132			30.772	35.102	31.847	36.37	35.918
52	43.227	40.816	35.339	30.248	35.14	42.48	44.089				41.761		39.192	40.824		40.033			30.977							45.722	39.192	35.918	41.625	40.947
53	42.425	43.265	45.78	41.705	46.645	35.102	37.551				32.663		36.744	37.311		37.56			34.334							36.744	43.273	40.008	35.32	35.955
54	46.437	48.163	43.641	37.631	40.008	40.824	44.082				37.772		37.693	47.347		39.26			31.192							40.816	37.56	39.218	39.808	40.816
55	43.542	40.849	46.88	41.633	41.641	38.402	35.102				44.965		38.506	43.273		39.26			29.6021667							35.1664074	40.816	31.837	38.808	35.112
56	45.083	42.48	33.509	35.704	42.457	38.367	35.928				42.48		38.445	44.898		36.88											43.296	26.951	37.462	42.142
57	39.724	45.722	40.406	38.445	45.831	40	49.856				40.947		35.928	36.744		31.02											38.445	32.097	40.947	38.506
58	38.445	41.633	46.466	36.002	43.265	42.457	39.252				34.286		50.619	38.402		39.489											38.367	35.14	36.988	33.028
59	40.947	43.273	45.714	40.39	42.457	43.296	40.816				38.402		31.031	44.15		35.955											42.48	28.618	35.564	38.376
60	53.086	46.538	36.735	39.192	46.595	41.641	41.633				39.26		28.676	35.955		31.031											50.639	37.56	36.15	36.735
61	46.301	45.722	37.176	40.53	49.007	40.824	50.612				49.796		35.928	44.965		40.849											40.033	34.286	43.48	27.803
62	49.963	40	32.816	34.266	39.192	40.008	38.367				40.008		41.705	42.449		36.771											35.102	40.008	39.674	50.036
63	42.731	43.265	38.402	42.613	40.824	35.102	34.451				39.192		37.5186613	36.771		38.376											47.347	30.215	35.187	41.761
64	44.187	44.082	42.48	46.437	40	35.112	39.26				40.89			41.641		39.3203651											40.299	33.509	36.916	42.386
65	41.335	40.824	37.551	29.569	29.399	42.457	41.832				35.102			45.78													33.479	34.295	29.308	42.449
66	41.761	41.633	40.033	35.112	46.538	41.665	44.202				42.48			45.743													40.849	38.445	30.116	40
67	44.905	42.457	43.296	34.286	36.744	45.714	45.722				39.26			36.816													44.15	33.509	42.355	38.376
68	51.429	43.273	38.61	35.928	42.52	47.354	39.184				40.849			40.947													42.52	39.184	35.779	36.735
69	46.709	35.112	30.204	45.831	36.735	50.639	32.694				39.218			48.191													38.445	45.743	35.14	35.112
70	40.299	48.225	33.149	35.112	42.449	37.56	40.816				47.41			43.265													36.744	43.296	38.428	47.459
71	44.353	42.449	38.376	37.693	44.082	37.551	41.109				48.191			37.586													32.653	31.031	32.097	44.928
72	51.487	43.296	37.462	36.735	41.633	52.072	35.918				39.218			40.89													43.273	36.662	49.963	38.506
73	42.964	43.273	39.319	34.286	37.551	50.619	47.347				44.112			36.002													41.633	32.694	43.93	32.622
74	40.996	35.918	37.06	33.469	40.008	46.531	35.187				32.653			32.663													40	39.396	36.961	35.639
75	32.745	34.286	39.319	46.595	42.449	48.98	47.347				36.744			48.163													44.898	34.295	38.791	38.919
76	39.598	39.184	36.002	35.102	35.187	42.3274133	43.204				36.735			41.633													38.376	35.14	36.662	44.36
77	42.574	38.376	42.449	39.26	35.112		45.743				51.487			32.816													40.0179342	37.586	36.662	42.855
78	46.595	35.928	41.633	33.469	29.388		35.112				39.218			39.319														34.286	37.462	36.37
79	45.78	40.033	34.325	37.772	41.02		51.429				45.78			42.449														36.735	38.367	41.569
80	34.101	41.641	44.089	36.816	36.771		44.082				40.849			35.112														40.008	35.955	28.757
81	44.112	42.457	41.109	38.402	49.796		46.987				42.449			36.744														36.735	45.341	36.443
82	45.37	41.641	38.402	33.509	35.955		36.662				43.273			45.722														34.441	41.384	46.07
83	51.429	35.928	40.075	45.831	42.449		48.225				48.986			32.653														34.902	46.645	39.598
84	51.377	28.571	36.066	31.847	35.928		49.823				39.218			39.184														35.3421325	39.022	39.43
85	43.204	45.714	36.744	29.263	46.538		40.824				39.192			41.832															34.022	37.816
86	43.755	47.354	35.955	35.339	44.905		35.928				36.744			38.402															39.724	44.353
87	42.52	41.641	43.265	33.717	46.559		43.265				33.479			40.89															34.873	46.178
88	48.191	41.665	35.639	40.89	51.487		41.641				41.641			44.15															45.671	31.117
89	42.948	44.089	35.244	36.744	44.082		42.52				42.457			55.606															37.06	32.694
90	40.53	27.767	40.849	41.641	53.061		41.641				40.033			45.743															45.692	38.6407416
91	49.796	22.056	38.713	48.163	44.928		39.218				28.583			37.631															38.919	
92	46.178	28.571	36.834	40.824	38.367		41.641				37.56			37.631															41.832	
93	43.885	41.641	40.816	45.743	34.295		44.089				47.375			40.824															39.396	
94	47.298	36.735	40.033	40.824	40.4583118		49.224				41.633			35.928															45.714	
95	40.824	38.367	34.325	36.735			41.025617				43.296			35.928															39.218	
96	35.928	40.816	44.27	40.816							44.112			39.396															27.136	
97	31.02	42.457	36.735	45.722							40.89			47.354															37.693	
98	48.225	41.665	43.273	50.639							44.089			29.433															40.53	
99	47.354	36.002	41.641	44.27							40.133			39.192															45.083	
100	37.631	38.367	40.033	37.869							46.595			41.214															43.885	
101	49.149	40.00242	37.56	40.8							48.191			40.406															42.574	
102	46.207		33.469	38.367							43.265			29.569															41.02	
103	36.37		43.296	39.758							36.771			40.008															44.928	
104	41.384		39.218	44.15							34.286			40															39.2767087	
105	40.824		40.008	49.655							38.115			44.831																
106	40.947		37.693	39.252							43.273			30.215																
107	50.355		42.52	41.625							46.987			39.724																
108	44.928		24.49	42.449							48.98			40.075																
109	38.713		45.78	32.673							39.184			45.157																
110	37.56		44.089	31.063							30.215			41.705																
111	43.296		40.849	39.226							34.325			38.7294909																
112	35.863		40.033	34.412							35.955																			
113	47.459		40.816	36.039							36.816																			
114	39.497		40.008	38.115							28.618																			
115	47.354		43.335	33.469							33.469																			
116	37.746		38.376	31.795							35.187																			
117	51.713		39.3599138	31.763							37.56																			
118	42.237			36.066							35.102																			
119	49.823			30.204							39.26																			
120	46.092			32.5							45.743																			
121	52.404			37.23							44.15																			
122	43.1412149			40.324							40.299																			
123				41.641							35.928																			
124				42.48							26.951																			
125				39.218							31.288																			
126				37.551							42.457																			
127				38.367							45.831																			
128				46.645							40.849																			
129				36.961							37.586																			
130				34.325							44.112																			
131				37.586							42.48																			
132				33.509							32.653																			
133				37.176							43.265																			
134				43.273							35.955																			
135				47.354							40.133																			
136				41.335							41.109																			
137				35.928							44.082																			
138				42.48							44.965																			
139				45.976							37.666																			
140				51.487							37.06																			
141				44.15							32.694																			
142				43.45							42.731																			
143				49.312							31.847																			
144				36.834							41.633																			
145				42.142							42.457																			
146				65.23							41.705																			
147				39.218							41.641																			
148				38.367							32.948																			
149				44.69							39.064																			
150				42.832							40																			
151				45.743							45.961																			
152				50.717							47.375																			
153				50.619							41.633																			
154				47.241							35.102																			
155				44.353							36.039																			
156				44.69							42.48																			
157				46.559							39.022																			
158				35.928							30.566																			
159				45.26							39.064																			
160				36.232							37.132																			
161				37.176							39.598																			
162				54.032							34.325																			
163				37.631							33.628																			
164				33.149							41.92																			
165				34.295							29.972																			
166				38.9699576							33.559																			
167											33.954																			
168											32.653																			
169											36.771																			
170											34.101																			
171											39.26																			
172											40.947																			
173											36.443																			
174											39.319																			
175											44.248																			
176											43.103																			
177											38.254																			
178											49.149																			
179											36.15																			
180											59.91																			
181											51.403																			
182											47.523																			
183											44.45																			
184											38.376																			
185											38.506																			
186											39.252																			
187											39.396																			
188											36.26																			
189											41.705																			
190											43.48																			
191											34.295																			
192											34.295																			
193											34.653																			
194											32.653																			
195											36.37																			
196											35.168																			
197											36.735																			
198											43.103																			
199											39.022																			
200											28.757																			
201											40.996																			
202											37.382																			
203											31.931																			
204											38.376																			
205											38.263																			
206											33.747																			
											39.9484029																			
number of fi	121	102	118	165	93	75	94	40	30	20	206	48	62	110	26	63	48	46	54	24	14	30	50	32	35	54	76	83	103	89
	595					259					452					235					161					405				
average	43.1412149	40.00242	39.3599138	38.9699576	40.4583118	42.3274133	41.025617	38.570225	41.4033	42.1877	39.9484029	39.9040417	37.5186613	38.7294909	38.597	39.3203651	37.6751042	32.4206957	29.6021667	34.579625	34.6075	39.6837667	37.06132	36.1556563	37.9714857	35.1664074	40.0179342	35.3421325	39.2767087	38.6407416
	40.38636361					41.10285106					38.93951936					34.71959133					37.09594573					37.68878488				
