# Supplementary material for: Carbohydrate sulfotransferase 14 gene deletion induces dermatan sulfate deficiency and affects collagen structure and bowel contraction
Source: PLoS One. 2025 May 6;20(5):e0320943. doi: 10.1371/journal.pone.0320943 (PMC12054877; doi:10.1371/journal.pone.0320943)
Supplement: S7 Table — (PDF) [file pone.0320943.s013.pdf]

Body weight change rate (%)

	Chst14+$^{/+}$																Chst14$^{-/-}$													
day	day 8								day 15								day 8							day15						
1	0	0	0	0	0	0	0	0	0	0	0	0	0	0	0	0	0	0	0	0	0	0	0	0	0	0	0	0	0	0
2	-1.7064846	1.77304965	0.90497738	2.13675214	1.26582278	-2.3569024	-1.384083	1.83823529	-4.0677966	-0.6734007	0	0.35211268	0.9375	-3.4351145	-1.4234875	-1.056338	-1.1320755	3.67346939	-1.6	2.48962656	4.76190476	-1.2195122	2.94117647	-0.8695652	0.37174721	0	2	-1.5748031	0.71684588	2.09205021
3	-3.4129693	3.19148936	0.45248869	2.56410256	2.53164557	-2.6936027	-1.384083	-0.3676471	-0.3389831	-0.3367003	1.01694915	3.16901408	-1.5625	0.38167939	-1.0676157	0.35211268	-2.6415094	0.40816327	-2	4.14937759	8.22510823	-0.4065041	1.96078431	-1.3043478	-0.3717472	0	1.2	-3.1496063	0.35842294	-0.41841
4	-2.3890785	3.54609929	0.45248869	2.56410256	1.26582278	-0.3367003	2.42214533	1.10294118	-0.3389831	1.01010101	-0.3389831	4.92957746	-5.9375	0.38167939	0	1.05633803	-0.3773585	1.2244898	-1.6	0.41493776	8.22510823	-2.8455285	0.49019608	-0.4347826	-0.7434944	1.6	0.4	-2.3622047	-1.4336918	2.92887029
5	-2.0477816	4.96453901	1.80995475	2.13675214	2.10970464	-2.020202	1.38408304	0.73529412	1.01694915	2.35690236	3.72881356	8.8028169	-6.875	1.90839695	-0.3558719	-0.3521127	1.13207547	5.30612245	-0.8	3.31950207	7.35930736	-1.2195122	3.43137255	0	0.37174721	2	4	-2.3622047	-0.7168459	2.51046025
6	-3.4129693	2.4822695	1.35746606	3.41880342	2.53164557	-3.7037037	-1.384083	-0.7352941	-2.0338983	0.33670034	2.71186441	10.2112676	-7.1875	2.67175573	-2.8469751	3.16901408	-0.754717	1.2244898	-2.8	4.97925311	4.76190476	-0.4065041	-0.4901961	-0.4347826	-0.3717472	0	3.6	-3.5433071	0	4.18410042
7	-5.8020478	2.12765957	3.16742081	0.42735043	2.10970464	-6.7340067	-2.0761246	1.47058824	-2.3728814	-0.6734007	1.01694915	5.98591549	-8.125	1.90839695	-3.5587189	2.46478873	-2.2641509	2.04081633	-2.8	7.88381743	4.32900433	-4.4715447	0	0	-4.8327138	-0.8	2	-2.7559055	-0.7168459	0.41841004
8	-7.8498294	-0.7092199	7.239819	-1.7094017	5.06329114	-7.7441077	-0.3460208	1.47058824	-1.0169492	-3.7037037	-0.6779661	5.63380282	-10	5.72519084	-4.6263345	2.46478873	-3.7735849	1.63265306	-2	9.54356846	10.3896104	-1.2195122	-1.4705882	1.30434783	-5.204461	0.4	0.4	-7.0866142	-4.6594982	-2.0920502
9									-1.0169492	-14.141414	-6.779661	2.46478873	-17.5	1.52671756	-5.6939502	1.05633803								2.60869565	-5.5762082	2.4	-3.2	-7.0866142	-7.8853047	-2.0920502
10									-2.3728814	-10.43771	-13.559322	3.16901408	-20	2.67175573	-4.9822064	0.35211268								3.47826087	-5.5762082	2	-0.8	-6.6929134	-27.240143	-0.8368201
11									2.37288136	-7.4074074	-15.932203	5.63380282	-21.25	3.81679389	-1.0676157	5.98591549								3.04347826	-3.3457249	3.6	4	-2.3622047	-3.5842294	2.09205021
12									5.08474576	-2.3569024	-11.525424	6.69014085	-12.5	4.58015267	-1.0676157	6.69014085								5.65217391	-2.2304833	4.8	3.6	-1.9685039	-1.0752688	4.18410042
13									5.08474576	-0.3367003	-4.7457627	3.16901408	-9.375	5.72519084	1.42348754	7.04225352								4.7826087	2.23048327	7.6	6.4	1.57480315	1.07526882	0.41841004
14									6.10169492	-0.6734007	-3.3898305	2.11267606	-7.8125	6.10687023	2.13523132	8.09859155								5.2173913	-1.1152416	6.4	7.2	1.96850394	3.58422939	4.18410042
15									7.79661017	7.74410774	0	5.63380282	-1.875	11.8320611	1.77935943	11.971831								8.69565217	5.20446097	10	7.6	6.2992126	4.30107527	4.18410042

DAI score

	Chst14$^{+/+}$																Chst14$^{-/-}$													
day	day 8								day 15								day 8							day15						
1	0	0	0	0	0	0	0	0	0	0	0	0	0	0	0	0	0	0	0	0	0	0	0	0	0	0	0	0	0	0
2	0	0	0	0	0	0	0	0	1	0.5	0.5	0.5	0.5	0.5	0	0	0	0	0	0	0.5	0	0	0	0	0.5	0	0	0	0
3	0	0	0	0	0	0	0	0	1.5	1.5	1	0.5	0.5	0.5	0	0	0	0	0	0	1.5	0	0	0.5	0	0.5	0	1	0	0
4	0	0	0	0	0	0	0	0	1	1.5	1	1	1	0	0	0	0	0	0	0	2	0.5	2	1	1	0.5	0	1	0.5	0
5	1	1	1	1	0	0	0	0	3	2.5	2.5	2	2	2	2	1	1	1	1	1	2.5	2	2	2	2	2	2	2	1	0
6	2	2	2	2	2.5	2.5	2.5	2.5	3	0	0	1	2	2	2	2	0	2	2	2	2.5	2	2	2	2	2.5	2	2	2	0.5
7	2	2	2	2.5	2	1	2	2	3	2.5	2	2.5	3	2.5	2	2	1	2.5	2	2	2.5	2.5	2	2.5	2	2.5	2	2	2.5	2
8	2.5	2	2	2.5	2	2	2	2	3	2.5	2.5	2.5	3	2	2	2	2	2.5	2	2	3	2.5	3	2.5	2	2.5	2	2	2.5	2.5
9									2.5	3.5	3	1	3.5	1	3	1								1	2	1	2.5	3	3	2.5
10									1.5	3	3.5	1	3.5	0	2.5	1								2	2	1	1	3.5	3	1
11									0	2	3.5	0	4	0	2.5	1								0	1.5	0	1	1.5	2.5	0
12									0	1	2.5	0	1.5	0	0.5	0								0	1.5	0	0	1.5	1.5	1
13									0	1	1.5	0	2	0	0	0								0	1	1	0	1	1	0
14									0	1	0.5	0	2	0	0	0								0	1	0	0	0	0	0
15									0	1	0	0	1.5	0	0	0								0	1	0	0	1	0	0

Water intake					
control		*day 8*		*day 15*	
Chst14+/+	*Chst14-/-*	*Chst14+/+*	*Chst14-/-*	*Chst14+/+*	*Chst14-/-*
5.46666667	5.16666667	7.15	6.51666667	5.8	5.11666667
5.55	4.48333333	7.18333333	6.98333333	7.73333333	5.9
5.21666667	6.55	6.95	5.31666667	6.48333333	5.53333333
5.93333333	4.98333333	5.76666667	6.1	6.85	6.48333333
7.93333333	4.86666667	5.86666667		8.43333333	6.25
5.68333333	5.7	5.63333333		7.16666667	6.05
5.46666667	4.51666667			6.16666667	5.71666667
5.83333333				5.95	

Colon length of DSS colitis models					
control		*day 8*		*day 15*	
Chst14+/+	*Chst14-/-*	*Chst14+/+*	*Chst14-/-*	*Chst14+/+*	*Chst14-/-*
83.053	72.632	59.234	62.138	75.785	68.921
74.957	64.501	56.882	59.89	65.314	73.683
72.923	70.894	62.25	60.692	68.81	66.12
77.482	63.31	51.276	69.016	77.661	60.486
74.634	64.017	56.383	57.035	66.503	68.151
90.363	63.902	66.181	66.319	78.196	76.238
80.757	54.979	60.7	47.749	64.385	74.763
89.272		61.437		84.133	

H&E score					
control		*day 8*		*day 15*	
Chst14+/+	*Chst14-/-*	*Chst14+/+*	*Chst14-/-*	*Chst14+/+*	*Chst14-/-*
0	0	13	13	3	3
0	0	13	4	12	12
0	0	9	3	14	3
0	0	13	10	14	6
0	0	13	13	13	4
0	0	14	12	12	7
0	0	13	13	12	7
0		13		4	
