# Supplementary material for: Carbohydrate sulfotransferase 14 gene deletion induces dermatan sulfate deficiency and affects collagen structure and bowel contraction
Source: PLoS One. 2025 May 6;20(5):e0320943. doi: 10.1371/journal.pone.0320943 (PMC12054877; doi:10.1371/journal.pone.0320943)
Supplement: S8 Table — (PDF) [file pone.0320943.s014.pdf]

Number of Ly-6G-positive cells measured										
genotyping	*Chst14+/+*					*Chst14-/-*				
group	control									
No.	*1*	*2*	*3*	*4*	*5*	*1*	*2*	*3*	*4*	*5*
1	0	0	0	0	0	0	0	0	0	0
2	0	0	0	0	0	0	0	0	0	0
3	0	0	0	0	0	0	0	0	0	0
average	0	0	0	0	0	0	0	0	0	0
group	day 8									
No.	*1*	*2*	*3*	*4*	*5*	*1*	*2*	*3*	*4*	*5*
1	114	106	255	475	245	245	136	39	0	945
2	148	103	271	464	256	234	130	53	0	847
3	150	114	230	453	241	240	128	44	0	876
average	137.333333	107.666667	252	464	247.333333	239.666667	131.333333	45.3333333	0	889.333333
group	day 15									
No.	*1*	*2*	*3*	*4*	*5*	*1*	*2*	*3*	*4*	*5*
1	149	746	380	280	129	200	0	0	52	135
2	123	774	403	275	139	221	0	0	54	144
3	165	745	412	294	148	232	0	0	54	120
average	145.666667	755	398.333333	283	138.666667	217.666667	0	0	53.3333333	133
