# Supplementary material for: Carbohydrate sulfotransferase 14 gene deletion induces dermatan sulfate deficiency and affects collagen structure and bowel contraction
Source: PLoS One. 2025 May 6;20(5):e0320943. doi: 10.1371/journal.pone.0320943 (PMC12054877; doi:10.1371/journal.pone.0320943)
Supplement: S2 Table — (PDF) [file pone.0320943.s008.pdf]

Gene expression of CS/DS synthase

Gene	*Chst3*		*Chst11*		*Chst12*		*Chst14*		*Chst15*		*Ust*		*Dse*	
Genotype	$Chst14^{+/+}$	$Chst14^{-/-}$	$Chst14^{+/+}$	$Chst14^{-/-}$	$Chst14^{+/+}$	$Chst14^{-/-}$	$Chst14^{+/+}$	$Chst14^{-/-}$	$Chst14^{+/+}$	$Chst14^{-/-}$	$Chst14^{+/+}$	$Chst14^{-/-}$	$Chst14^{+/+}$	$Chst14^{-/-}$
1	0.80464	0.770861	1.108794	1.018529	1.198702	0.902857	1.397831	0.002268	1.215314	0.972457	1.187558	0.721521	0.866978	0.752366
2	1.568418	0.828379	1.053385	0.803791	1.115511	0.804598	0.936586	0.007045	0.953457	0.823648	1.093781	0.75512	1.028875	0.797606
3	0.633945	0.846115	0.571369	0.950899	0.368588	0.579515	0.49691	0.000888	0.449226	0.617233	0.408858	0.87426	0.63519	0.739919
4	1.198895	0.938797	1.176155	1.027522	1.172817	0.669808	1.076732	0.008242	1.237662	0.899954	1.466487	1.197926	1.367243	0.803294
5	0.736871	0.791498	0.89829	1.065619	0.905108	0.99215	0.908612	0.01122	1.100216	0.866991	0.842856	1.134729	0.912915	0.942865
6	1.057231	0.860619	1.192007	0.994507	1.239274	0.601486	1.183329	0.01054	1.044125	0.642695	1.000461	0.937623	1.188799	0.889054

Gene expression related to collagen-related factors

Gene	*Col1a1*		*Col3a1*		*Dcn*		*Bgn*		*Vcan*	
Genotype	$Chst14^{+/+}$	$Chst14^{-/-}$	$Chst14^{+/+}$	$Chst14^{-/-}$	$Chst14^{+/+}$	$Chst14^{-/-}$	$Chst14^{+/+}$	$Chst14^{-/-}$	$Chst14^{+/+}$	$Chst14^{-/-}$
1	0.849103	0.643225	0.80464	0.770861	1.280693	1.050869	0.928074	0.853177	0.722862	0.737912
2	1.471629	0.728775	1.568418	0.828379	1.140812	0.866829	1.360265	0.803844	1.155094	0.868835
3	0.473105	0.836934	0.633945	0.846115	0.587787	0.911933	0.521587	0.796307	0.719514	1.131365
4	1.414436	0.878991	1.198895	0.938797	1.182302	0.96957	1.1754	0.793715	1.59781	0.944712
5	0.693751	0.795914	0.736871	0.791498	0.837383	0.871943	0.872981	0.816595	0.75258	1.092164
6	1.097977	0.597428	1.057231	0.860619	0.971023	0.981391	1.141694	0.803744	1.052139	0.962614

Gene expression related to DSS colitis

Gene	*Tgfb*						*Tnfa*						*Il1b*					
Group	control		day 8		day 15		control		day 8		day 15		control		day 8		day 15	
Genotype	$Chst14^{+/+}$	$Chst14^{-/-}$	$Chst14^{+/+}$	$Chst14^{-/-}$	$Chst14^{+/+}$	$Chst14^{-/-}$	$Chst14^{+/+}$	$Chst14^{-/-}$	$Chst14^{+/+}$	$Chst14^{-/-}$	$Chst14^{+/+}$	$Chst14^{-/-}$	$Chst14^{+/+}$	$Chst14^{-/-}$	$Chst14^{+/+}$	$Chst14^{-/-}$	$Chst14^{+/+}$	$Chst14^{-/-}$
1	1.057166	0.788378	4.153831	2.029317	3.449614	1.988038	1.235587	1.105608	34.19319	19.92081	5.505934	4.429292	0.630062	0.793834	24.06779	7.169719	13.03414	3.247217
2	0.915733	0.856592	1.792797	2.243951	3.022256	1.783083	0.703986	1.170353	14.84037	11.94805	9.31718	1.698292	0.384411	0.614685	14.95724	3.257866	11.72187	2.981396
3	0.917213	0.979024	2.046567	1.958103	2.28846	1.276023	1.157547	3.507783	16.95663	13.20004	4.301822	1.119927	1.405755	0.438973	16.54637	13.50389	5.817009	4.602349
4	0.956859	0.797131	2.989711	2.330174	1.566816	1.369132	0.649032	0.826498	20.39759	7.142615	3.397386	4.654416	1.489775	0.438973	65.69251	8.692927	4.894888	3.379878
5	1.067845	1.005533	1.22353	0.729074	2.667817	1.124706	1.264132	0.456848	11.54695	3.702308	4.216277	3.650098	0.746895	0.650975	10.74501	4.388012	5.455787	4.210493
6	1.085184	0.77109	1.305936	3.522235	1.493217	1.245064	0.989716	0.770319	8.21141	35.81538	1.430028	2.574205	1.343102	1.110261	12.42707	45.86857	1.475889	1.854276

Gene expression related to colonal contraction

Gene	*Acta2*		*Myh11*		*Mlyk*		*Rock1*		*Ryr2*		*Kcnq1*		*Cacna1c*		*Cnn1*	
Genotype	$Chst14^{+/+}$	$Chst14^{-/-}$	$Chst14^{+/+}$	$Chst14^{-/-}$	$Chst14^{+/+}$	$Chst14^{-/-}$	$Chst14^{+/+}$	$Chst14^{-/-}$	$Chst14^{+/+}$	$Chst14^{-/-}$	$Chst14^{+/+}$	$Chst14^{-/-}$	$Chst14^{+/+}$	$Chst14^{-/-}$	$Chst14^{+/+}$	$Chst14^{-/-}$
1	1.16245	0.846318	1.269219	0.964266	1.202186	1.048902	1.001785	1.025162	1.432898	0.876946	1.049967	0.883066	1.405282	0.84224	1.084029	1.079018
2	1.447047	1.098956	1.423033	0.989197	1.405296	0.816176	1.228031	0.74084	0.973433	0.727115	0.873548	0.66392	1.240714	0.579408	1.30159	0.916066
3	0.347644	1.176761	0.11628	1.308879	0.189903	1.228822	0.558625	0.894686	0.156508	1.333439	0.723564	0.881435	0.174497	1.004818	0.265499	1.514591
4	1.231064	1.140078	1.470459	1.327692	1.439177	2.191206	1.741436	1.232434	1.47265	1.982676	1.284972	0.842772	1.318216	1.378975	1.665446	1.463293
5	0.759144	1.211964	0.696233	1.329729	0.774052	1.284828	0.786041	0.950608	1.042412	1.270719	0.839845	1.39061	0.96915	1.144295	0.7771	1.649302
6	1.052651	1.148581	1.024777	1.516354	0.989385	1.656707	0.684082	1.090068	0.922099	1.411392	1.228103	1.067656	0.892141	1.211978	0.906335	1.413888

Gene	*Calm1*		*Ano1*		*Adrb1*		*Adrb2*		*Chrm2*		*Chrm3*		*Chat*		*Htr1a*	
Genotype	$Chst14^{+/+}$	$Chst14^{-/-}$	$Chst14^{+/+}$	$Chst14^{-/-}$	$Chst14^{+/+}$	$Chst14^{-/-}$	$Chst14^{+/+}$	$Chst14^{-/-}$	$Chst14^{+/+}$	$Chst14^{-/-}$	$Chst14^{+/+}$	$Chst14^{-/-}$	$Chst14^{+/+}$	$Chst14^{-/-}$	$Chst14^{+/+}$	$Chst14^{-/-}$
1	0.825048	1.048754	1.060314	0.756682	1.58286	0.696052	1.52887	0.930305	0.994934	1.050118	1.622786	0.898512	0.931702	0.727287	2.604551	0.886269
2	1.056753	0.798715	0.948319	0.583387	0.725816	1.019827	0.859141	0.855694	1.2295	1.064581	1.171764	1.66809	1.320327	1.047869	0.513483	2.626768
3	0.787187	1.098489	0.576519	0.768597	0.293305	0.763531	0.507922	0.723046	0.184744	1.290799	0.375956	1.248679	1.043245	1.454612	0.840189	1.30543
4	1.706862	1.224805	1.530671	1.036488	1.040586	0.820076	1.2007	0.651939	1.687382	1.990949	0.979434	1.143133	1.32839	1.472898	0.173765	0.055974
5	0.728011	1.16631	0.935495	1.334786	1.116476	1.258257	1.057462	0.562657	0.846038	1.672065	0.653977	1.03527	0.622961	0.891525	0.716819	0.327529
6	0.896138	1.157062	0.948682	0.904696	1.240959	0.649165	0.845904	0.820084	1.057403	1.842361	1.196083	1.67194	0.753374	1.706908	1.151192	1.196276

Gene	*Htr2a*		*Htr2b*		*Htr3a*		*Htr3b*		*Htr4*		*Htr7*		*Cx26*		*Gja1*	
Genotype	$Chst14^{+/+}$	$Chst14^{-/-}$	$Chst14^{+/+}$	$Chst14^{-/-}$	$Chst14^{+/+}$	$Chst14^{-/-}$	$Chst14^{+/+}$	$Chst14^{-/-}$	$Chst14^{+/+}$	$Chst14^{-/-}$	$Chst14^{+/+}$	$Chst14^{-/-}$	$Chst14^{+/+}$	$Chst14^{-/-}$	$Chst14^{+/+}$	$Chst14^{-/-}$
1	1.944499	0.67892	0.686844	1.2075	1.307575	0.881724	1.206225	0.678252	0.949348	1.46001	1.431764	0.958246	1.164941	1.210754	0.931702	0.727287
2	0.642424	2.262009	1.237487	1.004201	1.082422	1.087675	0.951926	0.836789	1.102812	0.871459	0.628552	2.272458	0.93513	1.008076	1.320327	1.047869
3	0.804777	1.426695	0.51704	1.375316	0.282454	1.093948	0.145718	1.20232	0.602174	0.783724	0.963618	1.070578	0.831782	1.13316	1.043245	1.454612
4	0.49159	0.393001	1.103368	1.756135	1.325423	1.550814	1.408507	1.279558	1.479213	1.423756	0.673333	0.611262	1.307853	1.670371	1.32839	1.472898
5	1.031234	0.684224	1.225074	1.077303	0.916286	1.054839	1.026531	1.018303	0.760858	1.074158	1.072667	0.434159	0.780998	0.948571	0.622961	0.891525
6	1.085475	1.696367	1.230188	1.701649	1.08584	1.414585	1.261094	1.289191	1.105595	1.091048	1.230065	2.159826	0.979296	1.06413	0.753374	1.706908

Comparison of gene expression of collagen-related factors between middle-Aged and young mice

Gene	*Col1a1*				*Col3a1*				*Dcn*				*Bgn*				*Vcan*			
Group	young		middle aged		young		middle aged		young		middle aged		young		middle aged		young		middle aged	
Genotype	$Chst14^{+/+}$	$Chst14^{-/-}$	$Chst14^{+/+}$	$Chst14^{-/-}$	$Chst14^{+/+}$	$Chst14^{-/-}$	$Chst14^{+/+}$	$Chst14^{-/-}$	$Chst14^{+/+}$	$Chst14^{-/-}$	$Chst14^{+/+}$	$Chst14^{-/-}$	$Chst14^{+/+}$	$Chst14^{-/-}$	$Chst14^{+/+}$	$Chst14^{-/-}$	$Chst14^{+/+}$	$Chst14^{-/-}$	$Chst14^{+/+}$	$Chst14^{-/-}$
1	0.77532	0.745337	0.419559	0.569513	0.8719	0.898642	0.486719	0.60171	1.172785	0.799101	0.608264	0.784669	0.891716	0.904005	0.700881	0.817851	0.833308	0.919489	0.534295	0.758089
2	1.481368	0.608282	0.709418	0.657874	1.370853	0.834015	0.790637	0.867501	1.162554	0.597271	0.713621	0.826619	1.341577	0.729872	0.83822	1.066738	1.225987	0.774664	0.739208	0.597743
3	0.528201	0.786546	0.490598	0.517866	0.704748	0.857621	0.633839	0.344454	0.493531	0.749919	0.704361	0.140436	0.626044	0.791474	0.882964	0.785645	0.729659	1.099372	0.5612	0.227715
4	1.475591	0.847978	0.421646	0.511315	1.586047	1.29081	0.611787	0.675972	1.327268	0.838861	0.658581	0.765425	1.46252	1.237924	0.961024	0.916927	1.624224	1.120535	0.459688	0.665975
5	0.750798	1.142689	0.838641	0.472758	0.6192	0.789099	0.779397	0.631555	0.961902	1.133446	1.328415	0.706488	0.733754	0.764638	0.852063	0.926666	0.650055	0.961569	0.763479	0.538953
6	0.988722	0.666414	0.625651	0.799534	0.847252	0.867211	0.816142	1.041428	0.881958	0.813355	1.011523	1.058623	0.944389	0.838518	1.061559	1.101501	0.936767	1.002164	0.66912	1.321994

Comparison of gene expression of collagen-related factors between male and female mice

Gene	*Col1a1*				*Col3a1*				*Dcn*				*Bgn*				*Vcan*			
Group	male		female		male		female		male		female		male		female		male		female	
Genotype	$Chst14^{+/+}$	$Chst14^{-/-}$	$Chst14^{+/+}$	$Chst14^{-/-}$	$Chst14^{+/+}$	$Chst14^{-/-}$	$Chst14^{+/+}$	$Chst14^{-/-}$	$Chst14^{+/+}$	$Chst14^{-/-}$	$Chst14^{+/+}$	$Chst14^{-/-}$	$Chst14^{+/+}$	$Chst14^{-/-}$	$Chst14^{+/+}$	$Chst14^{-/-}$	$Chst14^{+/+}$	$Chst14^{-/-}$	$Chst14^{+/+}$	$Chst14^{-/-}$
1	0.969957	0.736931	1.108315	0.738337	0.791138	0.793989	0.85506	0.304624	1.300664	0.861276	1.083293	0.153913	0.994279	0.732949	1.025297	0.590625	0.815303	0.845037	0.757694	0.369158
2	1.561571	0.805442	1.375386	0.96051	1.382599	0.731724	0.917723	0.340114	1.098805	0.695412	1.279649	0.119146	1.218772	0.922603	1.218852	0.588296	1.178788	0.789774	0.887563	0.252974
3	0.613193	0.983568	1.071576	1.764888	0.695209	0.772598	0.817294	0.637104	0.626562	0.934989	1.029559	0.245597	0.606989	0.924416	0.899461	1.177317	0.795025	1.356107	0.837595	0.74791
4	1.444615	1.267439	1.420255		1.360224	1.014701	0.625078		1.224109	1.425198	0.308057		1.361154	0.933	1.127213		1.422485	1.551466	0.450714	
5	0.551711	0.875393	0.730394		0.716173	0.970518	0.487018		0.775139	0.775964	0.171664		0.8554	0.882216	0.903227		0.645213	0.865224	0.342443	
6	0.858953	0.48565			1.054657	0.541256			0.974722	0.830821			0.963406	0.61745			1.143186	0.789446		
