# Supplementary material for: Carbohydrate sulfotransferase 14 gene deletion induces dermatan sulfate deficiency and affects collagen structure and bowel contraction
Source: PLoS One. 2025 May 6;20(5):e0320943. doi: 10.1371/journal.pone.0320943 (PMC12054877; doi:10.1371/journal.pone.0320943)
Supplement: Attachment — Submitted filename: Response to Reviewers.docx [file pone.0320943.s017.docx]

Professor Nikos K Karamanos

Academic Editor

PLOS ONE

February 17, 2025

Dear Professor Karamanos,

Re: Resubmission of manuscript reference No. PONE-D-24-57016

We sincerely appreciate the opportunity to submit a revised version of our manuscript, entitled “Carbohydrate sulfotransferase 14 gene deletion induces dermatan sulfate deficiency and affects collagen structure and bowel contraction,” to PLOS ONE. We are deeply grateful for the time and effort that you and the reviewer have devoted to evaluating our work and for your constructive feedback, which has helped us improve the manuscript.

We have carefully considered all comments and revised the manuscript accordingly, incorporating the valuable suggestions provided by you and the reviewer. Below, we present our point-by-point responses to the comments, outlining the revisions we have made. All changes are highlighted in the revised manuscript. We hope that these modifications adequately address the concerns.

Editor’s comments

1. Please ensure that your manuscript meets PLOS ONE's style requirements, including those for file naming.

Response:
Thank you for your guidance. We have carefully reviewed and revised our manuscript to ensure that it adheres to PLOS ONE's style requirements, including proper file naming. Please find the updated files attached for your review.

2. To comply with PLOS ONE submissions requirements, in your Methods section, please provide additional information regarding the experiments involving animals and ensure you have included details on (1) methods of sacrifice, (2) and efforts to alleviate suffering.

Response:
Thank you for clarifying your comments and the necessary revisions. In accordance with *PLOS ONE*'s submission guidelines, we have updated the Methods section to include additional details regarding the animal experiments. Specifically, we have added the following information:

- Methods of sacrifice: The revised manuscript now states (*page 6, lines 105–108*):

*“In the present study, no invasive treatment was performed on the live mice. Animal welfare was closely monitored throughout the experiments, and no severe pain was observed in the mice. The mice were euthanized by cervical dislocation before sample collection.”*

3. Please review your reference list to ensure that it is complete and correct. If you have cited papers that have been retracted, please include the rationale for doing so in the manuscript text, or remove these references and replace them with relevant current references. Any changes to the reference list should be mentioned in the rebuttal letter that accompanies your revised manuscript. If you need to cite a retracted article, indicate the article’s retracted status in the References list and also include a citation and full reference for the retraction notice.

Response:
Thank you for your feedback. We have carefully rechecked the reference list to ensure that it is complete and accurate. We confirm that no retracted papers have been cited in this manuscript.
Additionally, the following changes have been made to the reference list:

- *Page 23, line 508*: Reference No. 41 has been added in relation to additional experiment.
- *Page 24, line 542*: Due to a citation error, the reference numbers have been corrected from 16 to 46.

These corrections have been incorporated into the revised manuscript, and the updated reference list has been adjusted accordingly.

Reviewer’ comments

Comment 1:
- In-depth Mechanistic Studies: The study could benefit from further mechanistic investigations to elucidate the specific pathways through which DS affects collagen assembly and colonic contraction. This could include exploring the molecular interactions between DS and collagen or other extracellular matrix components.

Response:
Thank you for your valuable suggestion. To clarify the mechanism by which DS affects collagen fibril assembly, we analyzed the GAG chains of decorin, a CS/DS proteoglycan, using Western blotting.

Specifically, we have added text and made revisions in the following sections:

- Materials and Methods: *pages 10–11, lines 210–230*
- Results: *pages 13–14, lines 281–291*
- Fig. 1F
- Figure legend: *page 15, lines 316–318*
- Discussion: *page 23, lines 506–512*

*“Biochemical alterations in GAGs resulting from the lack of DS lead to defective assembly of collagen fibrils in the skin [22]. DCN is known as a core protein of CS/DS-PG that binds to type 1 collagen [41]. In this study, DCN was identified as a core protein of CS/DS in the colon. Furthermore, DS on DCN in the colon was significantly reduced and replaced with CS in the absence of Chst14. Therefore, it was suggested that the mechanism of* *defective collagen fibril assembly in the colon is similar to that in the skin.”*

To investigate the mechanism of intestinal contraction, we examined the gene expression of intestinal contraction-related factors using RT-qPCR. However, significant differences were not observed. We have added:

- Discussion: *page 24, lines 526–530*

*“Intestinal contraction is affected by smooth muscle, nerves, ion channels, and cell adhesion. However, no differences were detected in the gene expression of the factors related to colon smooth muscle contraction (S1 Fig. A). There is a possibility that the structural change of collagen fibril assembly influences intestinal contraction, though further research is necessary.”*

- S1 Fig.
- Figure legend: *page 34, lines 762–777*

Comment 2:
- Broader Physiological Assessments: Additional experiments assessing other aspects of gastrointestinal function, such as motility patterns or responses to food intake, could provide a more complete picture of how DS deficiency affects the colon.

Response:
We attempted to evaluate intestinal peristalsis using ultrasound. However, difference of peristaltic movement of the colon was not detected between *Chst14*^+/+^ and *Chst14*^-/-^ mice.

- Discussion: *page 24, lines 533–536*

*“No differences in peristalsis were detected by abdominal ultrasound between Chst14^+/+^ and Chst14^-/-^ mice (S1, 2 movies, S4 Fig. A, B). These data indicate that there are no significant differences in the physiological function of the colon in these mice.”*

- S1, 2 Movie
- S4 Fig.

Furthermore, we attempted to perform an organ bath assay using the isolation method; however, we were unable to obtain accurate data due to the absence of a monitoring system and suboptimal experimental conditions.

Comment 3:
- Longitudinal Studies: Conducting longitudinal studies could help determine if the observed effects in *Chst14*^-/-^ mice change over time or with age, which may provide insights into the progression of symptoms that occur in patients with mcEDS.

Response:
We examined middle-aged male mice (36–40 weeks old) but did not observe any new phenotypes that developed with aging.

- Discussion: *page 25, lines 547–553*

*“Age-dependent increases in body weight and colon length were observed in middle-aged Chst14^+/+^ and Chst14^-/-^ mice compared with young mice (S2 Fig. A–C). The gene expression of Col1a1 was decreased in middle-aged Chst14^+/+^ and Chst14^-/-^ mice (S2 Fig. J). Similar to young mice, middle-aged Chst14^-/-^ mice also exhibited colon shortening compared with age-matched Chst14^+/+^ mice (S2 Fig. A, C). However, no age-related phenotypic changes were observed in the colons of middle-aged Chst14^-/-^ mice (S2 Fig. D–I).”*

- S2 Fig.
- Figure legend: *pages 34–35, lines 778–798*

Comment 4:
- Inclusion of Female Mice: The study primarily used male mice to avoid gender-related variations. Including female mice in future experiments could provide more comprehensive data and help understand any potential gender differences in DS function.

Response:
We added experimental data for female mice. Similarly to male mice, colon-shortening was observed in female *Chst14*^-/-^. **Gene expression of *Dcn* was significantly lower in female** *Chst14*^-/-^ **mice** compared to males.

- Discussion: *page 25, lines 554–560*

*“Female Chst14^-/-^ mice also showed reduced body weight and colon shortening, similar to male mice (S3 Fig A–C). Furthermore, nifedipine suppressed intestinal contractions and eliminated the difference in colon length between Chst14^+/+^ and Chst14^-/-^ mice (S3 Fig D). On the other hand, gene expression of Dcn, collagen-related factor, was significantly decreased in female Chst14^-/-^ mice compared with that in male Chst14^+/+^ and Chst14^-/-^ mice (S3 Fig F). Detailed examination of the physiological functions of the colon in female Chst14^-/-^ mice is a subject for future research.”*

- S3 Fig.
- Figure legend: *page 35–36, lines 799–814*

Comment 5:
- Comparative Studies with Human Tissue: Comparing the findings in mouse models with human tissue samples from mcEDS patients could validate the translational relevance of the animal model and refine the conclusions drawn from the study.

Response:
mcEDS is an extremely rare disease, and obtaining colon tissue from patients is difficult. As noted in our manuscript (page 22, lines 492–493), only **one** study has reported on the colon pathology of mcEDS patients. Given future opportunities, we intend to further investigate this topic.

Thank you for considering our revised manuscript. We look forward to your response. Should you require any further clarification, please do not hesitate to contact us.

Sincerely,

Takahiro Yoshizawa, PhD

Division of Animal Research, Research Center for Advanced Science and Technology, Shinshu University

3-1-1 Asahi, Matsumoto 390-8621, JAPAN

Phone: +81-263-37-2699

Fax: +81-263-37-2698

E-mail: tyoshizawa@shinshu-u.ac.jp
